# Advances in miRNA-Mediated Bidirectional Crosstalk and Immune Evasion Mechanisms Between Lung Cancer Cells and CD8^+^ T Cells

**DOI:** 10.3390/ijms27156918

**Published:** 2026-08-01

**Authors:** Xinyi Zhou, Tao Pang, Zhe Ge

**Affiliations:** School of Sports, Shenzhen University, Shenzhen 518060, China; 2020128254@email.szu.edu.cn (X.Z.); 2020128264@email.szu.edu.cn (T.P.)

**Keywords:** lung cancer, CD8^+^ T cells, immune evasion, extracellular vesicles, miRNAs

## Abstract

Lung cancer ranks first in both incidence and mortality among all malignancies, and tumor microenvironment (TME)-induced CD8^+^ T cell exhaustion is a critical factor driving immune evasion and compromising the efficacy of immunotherapy. MicroRNAs (miRNAs), as key post-transcriptional regulators, shuttle between lung cancer cells and CD8^+^ T cells via extracellular vesicles (EVs), serving as critical communication hubs that reshape the TME. This review systematically synthesizes recent literature to summarize the regulatory patterns of miRNAs on functions of lung cancer cells and CD8^+^ T cells, and dissect the molecular mechanisms underlying miRNA-mediated bidirectional crosstalk between these two cell types. This review focuses on the dual-pronged immune evasion strategy employed by lung cancer cells to counteract CD8^+^ T cells. On the one hand, lung cancer cells aberrantly express endogenous miRNAs, such as miR-20a, miR-149-5p, and miR-326, to remodel their surface ligands and establish immune camouflage. On the other hand, they actively secrete EVs enriched in specific miRNAs, including miR-7108-3p, miR-651-5p, and miR-24-3p, which directly suppress CD8^+^ T cell function. Furthermore, lung cancer cells secrete additional miRNAs, notably miR-6794-5p, miR-708-5p, and miR-1234-3p, to reprogram other TME components, namely tumor-associated macrophages (TAMs), natural killer (NK) cells, and myeloid-derived suppressor cells (MDSCs). These reprogrammed cells, in turn, indirectly attenuate CD8^+^ T cells through a relay-like mechanism via immunosuppressive cytokines or surface checkpoint molecules produced by these cells. In addition, competing endogenous RNA (ceRNA) networks formed by long non-coding RNAs (lncRNAs) and circular RNAs (circRNAs) in lung cancer cells regulate miRNA activity at multiple levels, further impairing the immune effector functions of CD8^+^ T cells. Conversely, activated CD8^+^ T cells also secrete miRNA-containing EVs, which deliver these miRNAs to tumor cells, thereby inhibiting tumor progression. Elucidation of this miRNA-based bidirectional communication network will not only advance our understanding of immune evasion mechanisms in lung cancer but also provide novel insights into cell-free immunotherapeutic approaches based on CD8^+^ T cell-derived vesicles.

## 1. Introduction

Lung cancer ranks first globally in both incidence and mortality among all malignancies, accounting for 18.7% of total cancer-related deaths [1]. Pathologically, lung cancer primarily encompasses small cell lung cancer (SCLC) and non-small cell lung cancer (NSCLC), with NSCLC including lung squamous cell carcinoma (LUSC), large cell carcinoma of the lung (LCC), and lung adenocarcinoma (LUAD) [2]. Although targeted therapies and immunotherapies have improved survival in some patients with advanced NSCLC, raising the 5-year survival rate to 24–31% [3], the high heterogeneity of the tumor microenvironment (TME) remains a bottleneck that limits treatment efficacy [4]. The TME is not merely a collection of cancer cells, but is composed of various immune cells, stromal cells, and the extracellular matrix. In lung cancer immune surveillance, CD8^+^ T cells play a crucial role as key effector cells of the adaptive immune system. As lung cancer progresses, however, cancer cells employ various mechanisms to downregulate activating receptors on the surface of CD8^+^ T cells and inhibit the release of cytotoxic granules, such as perforin and granzyme B, ultimately leading to effector exhaustion.

miRNAs are a class of endogenous non-coding small RNAs approximately 22 nucleotides in length. As potent post-transcriptional regulators, they play dual roles in lung cancer—either promoting or suppressing tumor growth [5]. More importantly, miRNAs can shuttle between lung cancer cells and CD8^+^ T cells via extracellular vesicles (EVs) [6,7], acting as signaling “messengers” to reshape the local immune status of the TME. It is worth noting that this miRNA-mediated intercellular communication is not a unidirectional process. CD8^+^ T cells can also use EVs to transmit their own miRNAs back to tumor cells [8], forming a “bidirectional crosstalk” network that inhibits cancer progression. Based on this, this article systematically reviews the latest research progress on miRNA-mediated remodeling of the lung cancer microenvironment, focusing on the molecular mechanisms underlying bidirectional crosstalk between CD8^+^ T cells and lung cancer cells as well as immune evasion, with the aim of providing a theoretical basis for a deeper understanding of the dynamic evolution of the lung cancer microenvironment and the development of cell-free, EV-based targeted therapies derived from CD8^+^ T cells.

## 2. Regulation of CD8^+^ T Cell Function by miRNAs

### 2.1. Antitumor Functions of CD8^+^ T Cells

CD8^+^ T cells are the primary effector cells responsible for tumor recognition and elimination; their infiltration levels and functional status directly influence the efficacy of immunotherapy and patient prognosis [9]. In many cases of NSCLC, CD8^+^ T cell numbers are reduced, or their function is impaired, indicating tumor immune evasion [10,11]. The full activation of CD8^+^ T cells depends on the precise regulation of dual signaling pathways: The first signal is provided by the T cell receptor (TCR) recognizing antigenic peptides presented by major histocompatibility complex class I (MHC-I) molecules; the second signal primarily depends on the binding of the core costimulatory receptor CD28 to the ligands CD80/CD86 on the surface of professional antigen-presenting cells [12]. The activation of costimulatory signals is a critical hub mediating CD8^+^ T-cell proliferation, anti-apoptosis, the acquisition of cytotoxicity, and the formation of a memory phenotype. Aberrant expression or blockade of the second signal can directly lead to CD8^+^ T-cell inactivation; however, under sustained antigenic stimulation, even in the presence of co-stimulation, CD8^+^ T cells will gradually enter a state of exhaustion [12]. Therefore, the integrity of the second signal, together with sustained antigenic stimulation, determines whether CD8^+^ T cells will develop into functional effector cells or memory cells, or become inactivated or exhausted. CD8^+^ T cells exert immune surveillance and cytotoxic effects during tumor growth primarily through three mechanisms: First, the perforin–granzyme pathway, in which perforin forms pores in the tumor cell membrane, facilitating the efficient entry of granzyme into tumor cells and thereby triggering various intracellular death pathways [13]. Second, the Fas/FasL pathway, in which Fas—a cell death signaling surface molecule—binds to the Fas receptor on the tumor cell surface via the Fas ligand (FasL), which is highly expressed on the surface of CD8^+^ T cells, directly inducing apoptosis [13]. Third, the cytokine pathway. Interferon-γ (IFN-γ) and tumor necrosis factor-α (TNF-α), secreted by CD8^+^ T cells, not only act directly on tumor cells to induce senescence or death and upregulate MHC-I and programmed cell death ligand 1 (PD-L1), but also broadly regulate immune cells, stromal cells, and endothelial cells within the TME, thereby establishing a synergistic anti-tumor network [14].

### 2.2. miRNA-Mediated Regulation of CD8^+^ T Cell Effector Functions and Biological Behaviors

Aberrant miRNA expression can directly regulate the cytotoxic activity of CD8^+^ T cells. For example, exogenous let-7b can be internalized by CD8^+^ T cells, specifically inhibiting the expression of programmed death receptor 1 (PD-1); simultaneously, let-7b can also enter *Kras*-mutation-driven lung cancer cells, downregulating their PD-L1 [15]. Furthermore, following let-7b treatment, the proportion of effector and memory CD8^+^ T cells increased, reversing CD8^+^ T cell exhaustion and leading to increased secretion of IFN-γ and granzyme B [15]. Conversely, some miRNAs directly attenuate CD8^+^ T cell activity; for example, miR-23a specifically targets and inhibits BLIMP-1 in CD8^+^ T cells, thereby suppressing the expression of granzyme B and IFN-γ and attenuating the cytotoxic function of CD8^+^ T cells [16]. For more details on the molecular mechanisms by which miRNAs regulate the effector functions and biological behavior of CD8^+^ T cells, see Table 1. In addition to acting directly on CD8^+^ T cells themselves, miRNAs can also indirectly amplify the antitumor efficacy of CD8^+^ T cells by modulating helper T cells. For example, the loss of miR-7 lifts its inhibition of MAPK4, activates the AKT/ERK/NF-κB pathway, and thereby promotes the polarization of CD4^+^ T cells toward Th1; furthermore, these polarized CD4^+^ T cells not only directly enhance antitumor immune responses but also recruit and activate CD8^+^ T cells [17].

### 2.3. Regulation of miRNAs in CD8^+^ T Cells by the ceRNA Network

miRNAs within CD8^+^ T cells are not regulated in isolation; their biological activity is precisely regulated by a competitive endogenous RNA (ceRNA) network composed of upstream long non-coding RNAs (lncRNAs). For example, *lncNDEPD1* acts as a “molecular sponge” in CD8^+^ T cells, binding to and inhibiting miR-3619-5p. This lifts the post-transcriptional repression of PD-1 by miR-3619-5p; the resulting upregulation of PD-1 expression then attenuates CD8^+^ T cell activity, thereby promoting immune evasion in lung cancer [18]. This ceRNA crosstalk mechanism, mediated by upstream lncRNAs, not only regulates CD8^+^ T cell activation but also renders the function of downstream miRNAs highly cell-type-dependent. Another example is miR-15a, which suppresses cell proliferation and survival in NSCLC cells, acting as a tumor suppressor [19]. However, in CD8^+^ T cells, lncRNA *Malat1* (which is highly expressed in NSCLC tissues) can serve as a molecular sponge to competitively sequester miR-15a, thereby relieving miR-15a-mediated repression of *CD28* and B-cell lymphoma-2 (*BCL2*). This in turn enhances CD8^+^ T cell activation, interleukin-2 (IL-2) production, and the formation of memory CD8^+^ T cells [20]. Although this study was validated in an infection model, it suggests that miR-15a suppresses T cell responses in CD8^+^ T cells and is functionally pro-tumorigenic in this context. Thus, miR-15a inhibits cancer cell proliferation in NSCLC cells and likewise suppresses T cell activation and survival in CD8^+^ T cells—its molecular function is consistent. However, because it operates in different cell types, the same molecular function leads to opposite biological outcomes: it is tumor-suppressive in cancer cells, whereas in CD8^+^ T cells it becomes pro-tumorigenic by dampening antitumor immunity.

**Table 1:** Regulatory Roles of miRNAs in the Effector Functions and Biological Behavior of CD8^+^ T Cells.

miRNA	Regulated Cells	Target	Biological Function	References
let-7b	CD8^+^ T cells; Tumor cells	PD-1; PD-L1	CD8^+^ T cells: PD-1 ↓, proportion of effector memory CD8^+^ T cells ↑, secretion of IFN-γ and granzyme B ↑; Tumor cells: PD-L1 ↓	[15]
let-7	CD8^+^ T cells	?	PI3K/AKT/mTORC2 ↓, ROS production ↓, terminal differentiation/exhaustion of CD8^+^ T cells ↓, memory cell formation ↑	[21]
miR-934	CD8^+^ T cells	SHP2	SHP2 ↓, secretion of IFN-γ, TNF-α, granzyme B, and perforin ↑	[22]
miR-23a	CD8^+^ T cells	BLIMP-1	BLIMP-1 ↓, granzyme B and IFN-γ ↓, cytotoxic function of CD8^+^ T cells ↓	[16]
miR-651-5p	CD8^+^ T cells	BCL2	BCL2 ↓, CD8^+^ T-cell apoptosis ↑	[7]
miR-7108-3p	CD8^+^ T cells	CD8A	CD8A ↓, CD107a ↓, decreased secretion of IFN-γ, perforin 1, and granzyme B	[6]
miR-20a-5p	CD8^+^ T cells	NPAT	NPAT ↓, decreased secretion of IFN-γ, TNF-α, perforin, and granzyme B	[23]
miR-301a	CD8^+^ T cells	Runx3	Runx3 ↓, accumulation of CD8^+^ T cells in the TME and IFN-γ secretion ↓, antitumor immune response ↓, lung cancer immune evasion and metastasis ↑	[24]
miR-15b	CD8^+^ T cells	DEDD	DEDD ↓, CD8^+^ T-cell apoptosis ↓; decreased expression of CD69 and secretion of IL-2 and IFN-γ, resulting in reduced activation and antitumor activity of CD8^+^ T cells	[25]
miR-15a	CD8^+^ T cells	CD28 and BCL2	CD28 and BCL2 ↓; CD8^+^ T-cell activation and IL-2 production ↓; memory cell formation ↓	[20]
miR-29; miR-130	Naive CD8^+^ T cells	miR-29: T-bet and Eomes; miR-130: IRF1 and IL6st [26]	miR-29: T-bet and Eomes ↓, CD62L ↑, activation markers CD69 and CD44 ↓, CD8^+^ T-cell proliferation ↓, secretion of Granzyme B, TNF-α, and IFN-γ ↓; miR-130: IRF1 and IL-6 secretion ↓, CD62L ↓, activation markers CD28 and CD69 ↑, PD-1 ↓, CD8^+^ T-cell proliferation ↑, secretion of perforin, granzyme B, TNF-α, and IFN-γ ↑	[27]
miR-181a-5p	CD8^+^ T cells	Id2	Id2 ↓, T-bet and Ifng ↓, IFN-γ secretion ↓	[28]
miR-629-5p	CD8^+^ T cells	IL2RB	IL2RB ↓, JAK-STAT signaling ↓, granzyme B ↓, reduced antitumor activity of CD8^+^ T cells	[29]
miR-27b-3p	CD8^+^ T cells	TOX	TOX ↓, PD-1 and TIM-3 ↓, reduced exhaustion of CD8^+^ T cells, and enhanced antitumor immune function ↑	[30]
miR-379-5p	CD8^+^ T cells	TIM-3, TIGIT	TIM-3 and TIGIT ↓, CD8^+^ T-cell exhaustion ↓, their cytotoxic function ↑	[31]
miR-149-3p	CD8^+^ T cells	PD-1 *, TIM-3 *, BTLA *, Foxp1 *	Downregulation of PD-1, TIM-3, BTLA, and Foxp1 inhibitory receptors; CD8^+^ T-cell exhaustion ↓	[32]
miR-29b; miR-198	CD8^+^ T cells	miR-29b: MCL-1; miR-198: JAK3	miR-29b: MCL-1 ↓, reduced anti-apoptotic capacity of CD8^+^ T cells; miR-198: JAK3 ↓, JAK3/STAT5/6 signaling ↓, CD8^+^ T-cell proliferation ↓	[33]
miR-31-3p	CD8^+^ T cells	?	CCDC137 and MTERF2 ↓, CD8^+^ T-cell proliferation and activation ↑	[34]
miR-491	CD8^+^ T cells	CDK4, TCF-1, Bcl-xL	CDK4, TCF-1, and Bcl-xL ↓; CD8^+^ T-cell proliferation and IFN-γ secretion ↓; apoptosis ↑	[35]
miR-150	CD8^+^ T cells	c-Myb	c-Myb ↓, Bcl-2 and Bcl-xL ↓, CD8^+^ T-cell survival ↓, memory cell formation ↓	[36]
miR-21	CD8^+^ T cells, CD4^+^ T cells	PTEN	PTEN ↓, Akt ↑, CD8^+^ T-cell proliferation and IFN-γ and IL-2 secretion ↑	[37]
miR-19b	CD8^+^ T cells	PTEN	PTEN ↓, FOXO/PI3K signaling ↑, CD8^+^ T-cell proliferation and secretion of IFN-γ and granzyme B ↑, apoptosis ↓	[38]
miR-330-3p	CD8^+^ T cells	CD244	CD244 ↓, CD8^+^ T-cell apoptosis ↓, effector function ↑	[39]
miR-155	CD8^+^ T cells	Ikbke, Bach1; Ship1	(1) Ikbke and Bach1 ↓, STAT1/IRF7-mediated type I interferon signaling ↓, CD8^+^ T-cell proliferation and IFN-γ secretion, memory cells ↑; (2) Ship1 ↓, pAkt ↑, Phf19 ↑, PRC2 ↑, CD8^+^ T-cell senescence and exhaustion ↓	[40,41]
miR-29	CD8^+^ T cells	IFN-γ	IFN-γ ↓, reduced effector function of CD8^+^ T cells	[42]
miR-23a/27a/24	CD8^+^ T cells	miR-23a: CD107a; miR-27a and miR-24: IFN-γ	CD107a ↓, IFN-γ ↓, cytotoxic function of CD8^+^ T cells ↓	[43]
miR-139 and miR-342; miR-150	CD8^+^ T cells	miR-139: perforin * and Eomes *; miR-150: CD25 *	miR-139 and miR-342: decreased perforin and Eomes, decreased cytotoxicity of CD8^+^ T cells; miR-150: decreased CD25 and IL-2 signaling	[44]
miR-519e-5p	CD8^+^ T cells	NOTCH2	NOTCH2 ↓, Notch signaling ↓, PD-1 ↑, granzyme B ↓, CD8^+^ T cell exhaustion ↑	[45]
miR-199a-5p	CD8^+^ T cells	MAGT1	MAGT1 ↓, magnesium ion influx and TCR/PLCγ1 signaling ↓, PD-1 ↑, NKG2D ↓, CD8^+^ T cell functional exhaustion ↑	[46]
miR-17-92	CD8^+^ T cells	TGFBR2; PTEN [47]	(1) TGFBR2 ↓, inhibitory effect of TGF-β1 ↓, IFN-γ production and antigen-specific cytotoxicity ↑; (2) PTEN ↓, mTOR ↑, CD8^+^ T cell proliferation ↑, granzyme B and KLRG1 ↑, CD127 ↓	[48,49]

Unless otherwise indicated, direct miRNA–target interactions were confirmed by luciferase reporter assays; * indicates targets predicted by in silico algorithms only; ? indicates that the target has not been clearly identified in the cited study. Arrows indicate the direction of change: ↑ represents upregulation, increased secretion, or enhanced function; ↓ represents downregulation, decreased secretion, or impaired function.

## 3. Inhibitory Effects of miRNAs Secreted by CD8^+^ T Cells on Tumor Cells

### 3.1. Mechanisms of EV miRNA Sorting, Delivery, and Target Specificity

Beyond serving as passive recipients of miRNA-mediated regulation, CD8^+^ T cells also actively participate in intercellular communication within the TME. Upon activation, CD8^+^ T cells can package their own miRNAs into EVs and deliver them to tumor cells or other TME components, thereby exerting direct or indirect antitumor effects. EVs carry a diverse repertoire of miRNA cargo, including both mature and precursor miRNAs, with the mature forms serving as the primary functional effectors upon delivery to recipient cells [50]. Importantly, miRNA incorporation into EVs is not a passive consequence of cellular abundance but is governed by selective sorting mechanisms. For instance, heterogeneous nuclear ribonucleoprotein A2/B1 (hnRNPA2B1) recognizes specific GGAG motifs in miRNA sequences and guides their loading into EVs, whereas synaptotagmin-binding cytoplasmic RNA-interacting protein (SYNCRIP) mediates the sorting of miRNAs containing GGCU motifs [50]. Once released, these EVs can transfer their miRNA cargo to recipient cells through multiple routes, including direct membrane fusion and various endocytic pathways [51], enabling the coordinated delivery of miRNAs along with cytotoxic proteins to exert combined post-transcriptional and protein-level regulatory effects on recipient cells. Beyond immediate and transient post-transcriptional repression, EV-delivered miRNAs can also exert long-term effects on recipient cells, particularly in modulating cell differentiation and expansion. For instance, specific miRNAs can drive tumor-associated macrophages (TAMs) polarization toward an M2 phenotype [52], promote the expansion of myeloid-derived suppressor cells (MDSCs) [53], and induce the differentiation of CD4^+^ T cells into regulatory T cells (Tregs) [54], as will be detailed in subsequent Section 5.3.

In the crowded TME, the ultimate destination of CD8^+^ T cell-derived EVs may be governed by a receptor-mediated “uptake competition.” EVs released from CD8^+^ T cells carry CD8 and TCR on their surface, enabling them to specifically recognize tumor cell-specific pMHC-I through TCR engagement [55]. Moreover, the coordinated action of TCR and CD8 ensures that the cytotoxic cargo carried within EVs is released into the immunological synapse only upon correct target cell recognition, thereby minimizing collateral damage to surrounding normal cells [55]. In addition, CD8^+^ T cell-derived EVs bear the adhesion molecules CD2 and LFA-1, which preferentially tether EVs to the tumor cell surface [55]. Beyond these receptor-mediated high-affinity interactions, tumor cells also exhibit robust macropinocytic activity, a receptor-independent, high-capacity endocytic process frequently exploited by tumor cells to scavenge extracellular nutrients in support of their rapid proliferation [56,57]. Although multiple cell types within the TME are capable of taking up CD8^+^ T cell-derived EVs, the combination of receptor-ligand-mediated high-affinity binding and the heightened macropinocytic activity of tumor cells predisposes lung cancer cells to preferentially capture CD8^+^ T cell-secreted EVs.

Although individual miRNAs are predicted to regulate hundreds to thousands of mRNAs, only a few signaling pathways are typically highlighted to explain the specific functions in T cell–tumor interactions. First, the degree of complementarity between the miRNA seed region and target sites determines binding affinity: high-affinity targets are preferentially silenced even at low miRNA concentrations [58], whereas low-affinity targets are repressed only when miRNAs are highly expressed. Second, the cellular context—i.e., the repertoire of target mRNAs actually expressed in a given cell type—dictates which predicted targets are available for regulation [59]. Third, a single miRNA can simultaneously target multiple nodes within the same signaling pathway, thereby achieving cooperative repression [60]. Fourth, target site accessibility can be modulated by RNA-binding proteins that compete with miRNAs for binding to the same 3′ UTR [61], a mechanism conceptually similar to the lncRNA-mediated ceRNA networks discussed in Section 2.3. Consequently, although the target spectrum of an individual miRNA is broad, the interplay of affinity hierarchy, cell type-specific expression context, and pathway convergence ensures that only relevant pathways are modulated in a given cellular setting.

### 3.2. EVs Secreted by CD8^+^ T Cells Directly Inhibit Tumor Cells

Although no study has yet shown whether CD8^+^ T cells can transmit miRNAs back to lung cancer cells, this active counter-regulatory mechanism has been demonstrated in other solid tumors. For example, CD45RO^−^CD8^+^ T cells are naive cytotoxic T cells, a subset of T lymphocytes that have not yet encountered specific antigens and remain in an unsensitized state. A study has shown that they can secrete miR-765-enriched small EVs (sEVs), which, upon delivery to endometrial cancer cells, downregulate proteolipid protein 2 (PLP2) to inhibit the Notch signaling pathway, thereby blocking epithelial–mesenchymal transition (EMT) and ultimately suppressing tumor progression [8]. Given that miR-765 can also inhibit the proliferation, migration, and invasion of A549 lung cancer cells [62], it is plausible that activated CD8^+^ T cells infiltrating the lung cancer microenvironment could similarly suppress malignant progression by secreting miR-765-enriched EVs. Furthermore, CD8^+^ T cell-derived EVs also carry granzyme B [63], whose cytotoxic action against lung cancer cells precedes the post-transcriptional regulation mediated by EV-encapsulated miRNAs. However, lung cancer cells can counteract granzyme B-mediated killing by overexpressing serine protease inhibitor B9 (Serpin B9) [64]. Consequently, once lung cancer cells successfully resist this initial acute protein assault, they enter a survival window during which EV-delivered antitumor miRNAs (such as miR-765) exert post-transcriptional control to suppress malignant progression. Together, these two phases form an “acute killing–sustained suppression” relay that jointly underpins the antitumor effector function of CD8^+^ T cells. Notably, the tumor-suppressive function of CD8^+^ T cell-derived vesicles is highly dependent on the cellular state of the donor T cells. sEVs secreted by activated CD8^+^ T cells effectively mediate antitumor effects; however, when CD8^+^ T cells enter an exhausted state, the expression profiles of miRNAs and lncRNAs packaged in their sEVs are significantly altered. These exhausted T cell-derived sEVs may not only lose their original tumor-suppressive capacity but also, upon uptake by healthy CD8^+^ T cells, in turn inhibit the proliferation, activity, and cytokine secretion of the recipient cells [65].

### 3.3. miRNA Secreted by CD8^+^ T Cells Regulates TME Cells to Synergistically Inhibit Tumors

CD8^+^ T cells can also reshape the stroma and immune cell clusters in the TME by secreting EVs, thereby exerting a synergistic antitumor effect. Studies have confirmed that activated CD8^+^ T cells derived from healthy mice can release EVs rich in miR-298-5p, which are laterally transferred to mesenchymal stromal cells and induce apoptosis, thereby clearing the stromal environment on which tumors depend for survival. This process has demonstrated significant antitumor effects in various solid tumor models, including fibrosarcoma, melanoma, and breast cancer [66]. However, CD8^+^ T cells derived from tumor-bearing hosts lose this ability: their EVs lack miR-298-5p and no longer possess the capacity to eliminate mesenchymal stromal cells [66], suggesting that the TME may, through some mechanism, impair the ability of CD8^+^ T cells to generate functional EVs. The discovery of this mechanism not only clarifies the molecular pathway by which CD8^+^ T cell extracellular vesicles degrade physical barriers in the stroma via specific miRNAs, but also lays an important theoretical foundation for further exploration of how tumors domesticate and suppress CD8^+^ T cell function. This indirect pathway, through which miRNA-rich EVs remodel stromal components of the TME to synergistically suppress tumors, not only broadens the scope of immune surveillance by effector T cells but also provides an important theoretical foundation for the future development of cell-free targeted immunotherapeutic strategies based on T cell-derived EVs.

## 4. Regulation of CD8^+^ T Cell Function by TME Cells

The TME is a complex ecosystem composed of cancer cells, immune infiltrates (including TAMs, MDSCs, Tregs, natural killer [NK] cells, and dendritic cells [DCs]), stromal cells such as cancer-associated fibroblasts (CAFs), and the extracellular matrix (ECM) [67]. In lung cancer, this microenvironment is predominantly immunosuppressive, characterized by the accumulation of pro-tumor immune cells and reduced infiltration or dysfunction of CD8^+^ T cells [68]. These cellular components do not act in isolation; rather, they form an interconnected regulatory network that coordinately suppresses CD8^+^ T cell-mediated anti-tumor immunity through multiple mechanisms, including inhibiting CD8^+^ T cell infiltration, impairing their activation and proliferation, suppressing the release of cytotoxic granules, and inducing exhaustion or apoptosis [68]. Understanding how other TME cell types promote CD8^+^ T cell dysfunction is essential for deciphering the EV-mediated immune evasion strategies discussed in subsequent sections.

### 4.1. TAMs

As the predominant immune cell population in the TME, TAMs are critically defined by their polarization state: they can be polarized into either an anti-tumor M1 or a pro-tumor M2 phenotype [69]. M2-like TAMs in the lung cancer microenvironment suppress the anti-tumor functions of CD8^+^ T cells by secreting IL-10 and TGF-β, disrupting metabolism, and upregulating immune checkpoint molecules [70]. Concurrently, they inhibit the production of the chemokines CXCL9 and CXCL10, thereby attenuating CXCR3-mediated chemotaxis of CD8^+^ T cells and reducing their recruitment into the TME [71].

### 4.2. MDSCs and Tregs

MDSCs are a heterogeneous population of immature myeloid cells that suppress the proliferation and cytotoxic activity of CD8^+^ T cells. Studies have demonstrated that highly expressed arginase-1 (Arg-1) and inducible nitric oxide synthase (iNOS) in MDSCs inhibit CD8^+^ T cell function by depleting L-arginine and producing nitric oxide, respectively: the Arg-1 pathway downregulates CD3ζ chain expression in CD8^+^ T cells, impairing their antigen recognition capacity, whereas the iNOS pathway inhibits CD8^+^ T cell proliferation and induces apoptosis [72].

Tregs are a critical immunosuppressive population. In the lung cancer microenvironment, Tregs not only suppress the secretion of effector molecules such as perforin, granzyme B, and IFN-γ by CD8^+^ T cells through abundant production of TGF-β and IL-10, but also exploit their surface CD25 (the interleukin-2 receptor α chain, IL-2Rα) to exert a “sponge effect,” competitively sequestering IL-2 that is essential for CD8^+^ T cell proliferation and activation [73].

### 4.3. NK Cells and DCs

Although NK cells and DCs are positive regulators of CD8^+^ T cell-mediated anti-tumor immunity, their functional impairment in the TME can indirectly compromise this immunity. Studies have demonstrated that NK cells promote the maturation and activation of conventional DCs through IFN-γ secretion, upregulating the expression of MHC-I, MHC-II, and the co-stimulatory molecules CD86 and CD40, thereby enhancing the antigen-presenting capacity of DCs and driving the proliferation and accumulation of stem-like CD8^+^ T cells in lung tissue [74]. Although this evidence originates from a breast cancer lung metastasis model, the mechanism by which NK cells promote DC maturation to drive CD8^+^ T cell responses appears broadly applicable across tumor types and may similarly operate in the lung cancer microenvironment.

### 4.4. CAFs

CAFs are the predominant stromal cell type in the TME and exert immunosuppressive effects on CD8^+^ T cells through both physical and biochemical mechanisms. At the physical level, CAFs remodel the ECM and deposit dense collagen fibers, constructing a physical barrier that restricts CD8^+^ T cell infiltration into tumor cell nests [75]. At the biochemical level, CAFs secrete TGF-β and activate the cyclooxygenase-2 (COX-2)/prostaglandin E2 (PGE2) signaling axis, both of which can suppress CD8^+^ T cell activation and IFN-γ production; concurrently, CAFs upregulate the immune checkpoint molecule PD-L1, promoting the exhaustion of infiltrated CD8^+^ T cells [75]. Through this dual physical and biochemical shielding, CAFs not only impede CD8^+^ T cells from accessing tumor cells but also functionally inactivate those that do reach the TME, thereby weakening the anti-tumor immune response.

In summary, diverse cell types within the TME construct a multidimensional immunosuppressive network through various mechanisms, including the secretion of soluble factors, immune checkpoint engagement, and metabolic competition. Notably, intercellular communication in this context does not rely solely on soluble mediators. EVs, as novel signaling vehicles, are increasingly recognized as indispensable communication hubs. The following sections will focus on how lung cancer cells utilize miRNA-containing EVs to actively educate other cells in the TME, thereby suppressing the anti-tumor functions of CD8^+^ T cells.

## 5. miRNA-Based Immune Evasion Strategies of Lung Cancer Cells Against CD8^+^ T Cells

Traditional tumor immunology holds that the infiltration of CD8^+^ T cells into tumor tissue is a prerequisite for the host to mount an antitumor immune response [76]. However, as tumors progress to a more malignant state, the pattern of CD8^+^ T cell infiltration often exhibits a complex and paradoxical nature. In the highly heterogeneous TME, the “spatial infiltration density” of CD8^+^ T cells does not equate to their “actual cytotoxic activity” [76]. To evade immune surveillance, tumor cells not only prevent CD8^+^ T cell infiltration by disrupting vascular endothelial adhesion and chemokine networks [77,78], thereby forming an “immune desert”-type escape; they can also target already infiltrated CD8^+^ T cells by regulating miRNA networks to blunt their effector functions, inducing them to become functionally impaired, exhausted, or even apoptotic [6,7], thereby achieving immune evasion in a context of “infiltration without killing.” Based on this, this chapter will systematically analyze the molecular mechanisms of immune evasion in lung cancer from the perspectives of CD8^+^ T-cell infiltration, the direct or indirect suppression of CD8^+^ T cells by miRNAs secreted by lung cancer cells, and the reshaping of surface ligands due to intrinsic miRNA dysregulation in tumors.

### 5.1. Infiltration of CD8^+^ T Cells into Tumor Tissue

In the dynamic evolution of the lung cancer microenvironment, CD8^+^ T cells in peripheral blood cannot directly exert antitumor effects. They must undergo two critical stages—crossing the vascular endothelial barrier and directed chemotaxis within the tumor stroma—before reaching lung cancer cell nests and forming immune synapses to exert cytotoxic effects. The core molecular mechanisms of this multidimensional migration process primarily encompass the following three stages:

Phase 1: Vascular endothelial recruitment and transendothelial migration. Attracted by local inflammatory factors, peripheral CD8^+^ T cells must first cross the vascular barrier to infiltrate. During this endothelial crossing process, adhesion molecules on vascular endothelial cells mediate an ordered cascade of events: E-selectin and VCAM-1 slow down CD8^+^ T cells in the bloodstream and cause them to roll along the vascular endothelium, while ICAM-1 primarily acts in concert with VCAM-1 to mediate subsequent firm adhesion. These three molecules act in an ordered sequence to collectively facilitate the endothelial crossing and infiltration of CD8^+^ T cells into tumor tissue (e.g., subcutaneous melanoma and peritoneal tumors) [79]. However, tumor-associated vasculature exhibits organ-specificity and heterogeneity: E-selectin is not expressed on pulmonary tumor vessels, whereas both VCAM-1 and ICAM-1 are expressed [79]. Therefore, in the lung cancer microenvironment, VCAM-1 and ICAM-1 serve as key adhesion molecules that mediate the transendothelial recruitment of CD8^+^ T cells. In vascular endothelial cells, miR-181b can inhibit the nuclear translocation of NF-κB by specifically targeting and inhibiting importin-α3, thereby suppressing the expression of VCAM-1, ICAM-1, and E-selectin [80]. Since lung cancer cells can secrete EVs rich in miR-181b [81], lung cancer cells may utilize EVs to deliver miR-181b to vascular endothelial cells, thereby suppressing the expression of key adhesion molecules and inducing a state of vascular endothelial dysfunction, which impedes the vascular recruitment and transendothelial infiltration of CD8^+^ T cells.

Phase 2: Spatially directed migration via the chemokine axis. Upon entering the tumor stroma, the precise infiltration of CD8^+^ T cells into lung cancer cell nests is highly dependent on navigational signals from the chemokine gradient network in the TME. In the lung cancer microenvironment, activated CD103^+^ classical dendritic cells and specific subsets of TAMs (such as the murine M3 and human M1^hot phenotypes) secrete high levels of the inflammatory chemokines CXCL9 and CXCL10 [77,78]. CD8^+^ T cells recognize this signal via the chemokine receptor CXCR3 on their surface, enabling them to migrate toward the tumor parenchyma along a concentration gradient [77]. However, this precise chemotactic navigation system is often disrupted during the progression of lung cancer. Studies have shown that miR-34a-5p is significantly overexpressed in peripheral blood T cells of lung cancer patients, and that this miRNA can target and degrade *CXCR3* transcripts by directly binding to the 3’UTR of *CXCR3* mRNA, thereby downregulating the CXCR3 protein on the surface of CD8^+^ T cells [82]. This finding suggests that, prior to the entry of CD8^+^ T cells into tumor tissue, miR-34a-5p-mediated downregulation of CXCR3 may have preemptively weakened the sensitivity of CD8^+^ T cells to chemokine signaling, thereby significantly reducing their subsequent efficiency of directed migration into the tumor stroma. In contrast to this negative blocking effect, artificial intervention with miRNAs can reverse the migration impairment. Studies have shown that miR-4664-3p is significantly overexpressed in NSCLC lung tissue. Specifically, downregulating *PRKCB* in NSCLC cells inhibits CD8^+^ T-cell infiltration and their effector functions, thereby exerting an immunosuppressive role in the TME and being associated with poor patient prognosis [83]. In a mouse model of NSCLC, intervention with a miR-4664-3p antagonist (antagomir, a chemically modified antisense oligonucleotide capable of competitively binding to and degrading miR-4664-3p) resulted in a significant increase in the number of CD8^+^ T cells infiltrating the tumor tissue, while the ability of CD8^+^ T cells to secrete IFN-γ was enhanced, thereby boosting the antitumor immune response and inhibiting tumor growth [83].

Stage 3: Immune synapse formation and final cytotoxic killing. When CD8^+^ T cells penetrate the stroma and successfully infiltrate the vicinity of lung cancer cells, the TCR on their surface specifically recognizes and binds to antigen peptides (pMHC-I) presented by MHC-I on the surface of lung cancer cells. With the cooperation of the CD8 co-receptor, an immune synapse forms at the interface between the two cell types [84]. This synaptic structure serves as the primary structural basis for the directed release of perforin and granzyme B by CD8^+^ T cells onto tumor cells, ensuring highly efficient and precise target cell killing [84]. It should be noted that CD8^+^ T cells can also release cytotoxic granules without forming a mature synapse, but the killing efficiency is only about one-third that of the synapse-mediated pathway [84]. However, the functional integrity of the immunosynapse is often compromised by miRNAs in the lung cancer microenvironment. For example, miR-7108-3p, secreted by lung cancer cells in sEVs, can specifically target and degrade *CD8A* in CD8^+^ T cells [6], thereby inhibiting the function of co-receptors on the T-cell side and suppressing the antitumor function of CD8^+^ T cells. At the same time, lung cancer cells can remodel their surface antigen-presenting molecules and co-inhibitory/co-stimulatory ligands through the aberrant expression of their own miRNAs, thereby disrupting the function of the immunological synapse on the tumor side and further impairing the recognition and killing capabilities of CD8^+^ T cells. The specific molecular mechanisms by which lung cancer cells remodel surface ligands via their own miRNAs to counteract CD8^+^ T cells will be elaborated in Section 5.4.2.

### 5.2. Lung Cancer Cells Secret miRNAs to Directly Inhibit CD8^+^ T Cells

Lung cancer cells directly suppress the immunological effector function of CD8^+^ T cells by actively secreting EVs rich in specific miRNAs. A study has shown that NSCLC cell-derived sEVs containing miR-7108-3p, upon uptake by CD8^+^ T cells, downregulate the surface degranulation marker CD107a and attenuate the production of effector molecules, including IFN-γ, perforin-1, and granzyme B [6]. Furthermore, in lung adenocarcinoma cells with epidermal growth factor receptor (*EGFR*) mutations, the EGFR signaling pathway activates the Fos proto-oncogene (*FOS*), promoting high expression of miR-651-5p; Subsequently, miR-651-5p is delivered to CD8^+^ T cells via sEVs, where it specifically downregulates *BCL2* in CD8^+^ T cells, thereby inducing apoptosis in these cells [7].

It is worth noting that certain miRNA-mediated immune evasion pathways are shared among different solid tumors [85], which provides important clues for uncovering unknown evasion mechanisms in lung cancer. A study has shown that miR-20a-5p-rich sEVs released by triple-negative breast cancer cells can be internalized by CD8^+^ T cells, thereby inhibiting their secretion of IFN-γ, TNF-α, perforin, and granzyme B, and reducing their ability to kill tumor cells [23]. Given that NSCLC cells can also secrete sEVs containing miR-20a-5p [86], this suggests that lung cancer cells can also deliver this miRNA to CD8^+^ T cells via sEVs, thereby inhibiting their antitumor function.

In addition to single miRNA delivery pathways, EVs derived from lung cancer cells can also carry upstream non-coding RNAs to establish complex ceRNA cascade regulatory networks within CD8^+^ T cells. A study has shown that sEVs secreted by NSCLC cells containing circUSP7, once taken up by CD8^+^ T cells, act as “molecular sponges” to bind and inhibit miR-934, thereby lifting its inhibition of SHP2 (Src homology protein 2 tyrosine phosphatase 2), and the resulting upregulation of SHP2 impairs CD8^+^ T cell function [22].

### 5.3. Lung Cancer Cells Secrete miRNAs That Remodel the TME, Indirectly Inhibiting CD8^+^ T Cells

Lung cancer cells not only directly suppress CD8^+^ T cells via miRNA-containing EVs, but also indirectly impair CD8^+^ T cell-mediated antitumor immunity by influencing other cells within the TME, including TAMs, MDSCs, Tregs, NK cells/DCs, and CAFs. Importantly, such effects are long-term: miRNA-mediated signaling drives the long-term differentiation and functional polarization of these other TME cells, thereby establishing a stable immunosuppressive microenvironment.

#### 5.3.1. TAMs

Lung cancer cells can also reprogram other cells in the TME by releasing miRNA-rich EVs, thereby indirectly suppressing the antitumor effects of CD8^+^ T cells. Among these mechanisms, the polarization of TAMs is a key indirect immunosuppressive mechanism. A study has shown that miR-6794-5p from A549-derived sEVs can be delivered to macrophages, where it activates the Janus kinase 1 (JAK1)/Signal Transducer and Activator of Transcription 3 (STAT3) pathway to induce M2 polarization, thereby suppressing the number and function of CD8^+^ T cells [87]; LUAD cells deliver miR-708-5p to TAMs via sEVs; this miRNA specifically inhibits phosphatase and tensin homolog (PTEN) in TAMs, thereby activating the protein kinase B (AKT)/mechanistic target of rapamycin (mTOR) pathway, driving M2 polarization while upregulating PD-L1 on the surface of TAMs, thereby indirectly inhibiting the antitumor function of CD8^+^ T cells [88]. Furthermore, miRNAs that drive M2 polarization in other solid tumors may also play similar roles in lung cancer. For example, hepatocellular carcinoma cells can release sEVs containing miR-23a-3p, which targets the PTEN/AKT pathway in TAMs and upregulates PD-L1 expression, thereby reducing the proportion of CD8^+^ T cells, decreasing IL-2 production, and promoting CD8^+^ T cell apoptosis [89]; colorectal cancer cells can secrete sEVs containing miR-1246, which, upon uptake by TAMs, drives their M2 polarization and further inhibits the infiltration and function of CD8^+^ T cells [90]. Notably, IGR-Heu lung cancer cells can also secrete EVs containing miR-23a-3p [91], and A549 cells can secrete sEVs carrying miR-1246 [92], suggesting that lung cancer cells may utilize M2-like TAMs by secreting EVs containing these miRNAs, thereby impairing the antitumor function of CD8^+^ T cells.

In addition to the unidirectional regulation of specific signaling pathways described above, lung cancer cells also utilize multiple miRNAs to synergistically regulate the TAM phenotype. The EVs they release can block TAM M1 polarization by delivering miR-146a [93], as well as deliver miR-3153 [52], miR-181b [81], miR-103a [94], miR-19b-3p [95], miR-21 [96], and miR-1290 [97] to drive the polarization of TAMs toward the M2 phenotype, shifting the M1/M2 balance toward an M2-dominant phenotype. These reprogrammed M2-like TAMs subsequently suppress CD8^+^ T cell infiltration and function via the mechanisms described in Section 4.1, including secretion of IL-10 and TGF-β, PD-L1 expression, and suppression of CXCL9/CXCL10 production [70,71]. Consequently, lung cancer cells can deliver miR-146a, miR-3153, miR-181b, and other miRNAs via EVs to reshape the polarization of TAMs, thereby suppressing the cytotoxic activity of CD8^+^ T cells through M2-like TAMs and enabling lung cancer to evade the immune system. Furthermore, previous studies have confirmed that various cancer cells, including those from laryngeal, prostate, and ovarian cancers, can respectively secrete sEVs containing miR-1246, miR-93-5p, and miR-205, to induce M2 polarization of TAMs [98,99,100]. Notably, A549 and H226 lung cancer cells have been shown to secrete sEVs carrying miR-1246 and miR-93-5p [92,101]. Clinical studies have also confirmed that sEVs derived from lung cancer cells in patients with LUAD are rich in miR-205 [102]. This suggests that lung cancer cells may induce M2 polarization of TAMs by secreting sEVs containing these miRNAs, thereby impairing the antitumor function of CD8^+^ T cells.

#### 5.3.2. MDSCs and Tregs

In addition, miR-21a from Lewis lung cancer cell-derived sEVs can induce the expansion of MDSCs by targeting and downregulating programmed cell death protein 4 (PDCD4) in bone marrow cells [53]. Expanded MDSCs suppress CD8^+^ T cell antigen recognition and proliferation and induce their apoptosis via the Arg-1 and iNOS pathways described in Section 4.2 [72]. Therefore, lung cancer cells can drive MDSC expansion by secreting miR-21a, thereby indirectly impairing the antitumor function of CD8^+^ T cells and recruiting Tregs. Moreover, studies in other tumor types have revealed similar sEVs miRNA-mediated regulatory mechanisms: breast cancer cells and glioma cells can secrete sEVs enriched with miR-9 and miR-1246, respectively, which are taken up by early MDSCs and CD14^+^ monocytes (the precursors of MDSCs), respectively, thereby driving the expansion, differentiation, and activation of MDSCs [103,104]. Given that lung cancer cells can secrete sEVs containing miR-9-3p and miR-1246 [92,105], it is suggested that lung cancer cells may, through these miRNAs, exploit MDSCs to indirectly impair the cytotoxic function of CD8^+^ T cells.

miR-214 from EVs derived from Lewis lung cancer cells can specifically inhibit *PTEN* in CD4^+^ T cells, driving the differentiation and expansion of this population of T cells into Tregs [54]. Reprogrammed Tregs subsequently suppress CD8^+^ T cell effector function and survival via the mechanisms described in Section 4.2, including secretion of TGF-β and IL-10, and CD25-mediated competitive sequestration of IL-2 [73]. Therefore, lung cancer cells can drive the phenotypic reprogramming of CD4^+^ T cells into Tregs by secreting miR-214, thereby indirectly impairing the antitumor function of CD8^+^ T cells.

#### 5.3.3. NK Cells and DCs

EVs secreted by hypoxic lung cancer cells are rich in miR-23a and miR-150-5p; when transferred to NK cells, they reduce the expression of CD107a and CD226 in NK cells, respectively, thereby reducing NK cell cytotoxicity [91,106]. Since NK cells can promote DC maturation via IFN-γ secretion, they in turn drive the proliferation and accumulation of CD8^+^ T cells [74]. Therefore, lung cancer cells may suppress NK cell cytotoxicity by secreting miR-23a and miR-150-5p, indirectly weakening the antitumor function of CD8^+^ T cells. In addition, miR-92b-3p in sEVs secreted by hepatocellular carcinoma cells, upon uptake by NK cells, can specifically inhibit the expression of the activation receptor CD69, thereby reducing NK cell cytotoxicity [107]. Given that SCLC cells can also secrete sEVs containing miR-92b-3p [108], this suggests that lung cancer cells may suppress NK cell function by secreting this miRNA, thereby weakening the antitumor function of CD8^+^ T cells.

In addition, tumor cells can regulate natural killer T (NKT) cells—a distinct T cell subset that expresses both TCR and NK cell receptors on their surface—via miRNAs. Studies have shown that miR-92a-3p produced by glioma cells can induce NKT cells to secrete large amounts of IL-6 and IL-10, thereby inhibiting the proliferation and activation of CD8^+^ T cells [109]. NSCLC cells also secrete miR-92a-3p [110], suggesting that lung cancer cells may utilize similar mechanisms to suppress CD8^+^ T cell function.

The NSCLC microenvironment can induce aberrant upregulation of miR-301a in DCs, thereby inhibiting DCs secretion of IL-12; the absence of IL-12 suppresses CD8^+^ T-cell activation and IFN-γ release, thus impairing the antitumor effects of CD8^+^ T cells [111]. Furthermore, in a Lewis lung cancer mouse model, specific knockout of the *Dicer1* gene in DCs—a gene whose absence blocks miRNA synthesis—delayed tumor growth [111]. However, the specific upstream mechanisms by which the microenvironment induces the upregulation of miR-301a in DCs remain unclear. This suggests that lung cancer cells can disrupt antigen presentation by interfering with miRNA expression in DCs, thereby indirectly impairing the cytotoxic activity of CD8^+^ T cells.

#### 5.3.4. CAFs

Not only do they target immune cells, but at the level of reprogramming stromal cells, miR-1290 and miR-210 secreted by lung cancer cells can specifically target and inhibit metallothionein 1G (*MT1G*) and TET methylcytosine dioxygenase 2 (*TET2*), driving the phenotypic transformation of normal lung fibroblasts (NLFs) or normal fibroblasts (NFs) into CAFs [112,113]. Reprogrammed CAFs subsequently restrict CD8^+^ T cell infiltration and suppress their function via the dual mechanisms described in Section 4.4, namely a physical barrier formed by ECM remodeling and biochemical inhibition mediated by TGF-β, the COX-2/PGE2 axis, and PD-L1 [75]. Consequently, lung cancer cells can use EVs to deliver miR-1290 and miR-210, thereby reprogramming NLFs into immunosuppressive CAFs and indirectly impairing the antitumor function of CD8^+^ T cells by exploiting this matrix barrier. Furthermore, melanoma cells secrete EVs rich in miR-155-5p and miR-92b-3p; upon uptake by NFs, these EVs induce reprogramming of NFs into CAFs [114,115]. Given that SCLC cells have been shown to transfer miR-92b-3p via sEVs [108], and Hcc827 lung cancer cells can also secrete sEVs rich in miR-155-5p [116], this suggests that lung cancer cells may induce the transformation of NFs into CAFs by secreting EVs containing miR-155-5p and miR-92b-3p, thereby indirectly suppressing the cytotoxic activity of CD8^+^ T cells.

**Figure 1:**
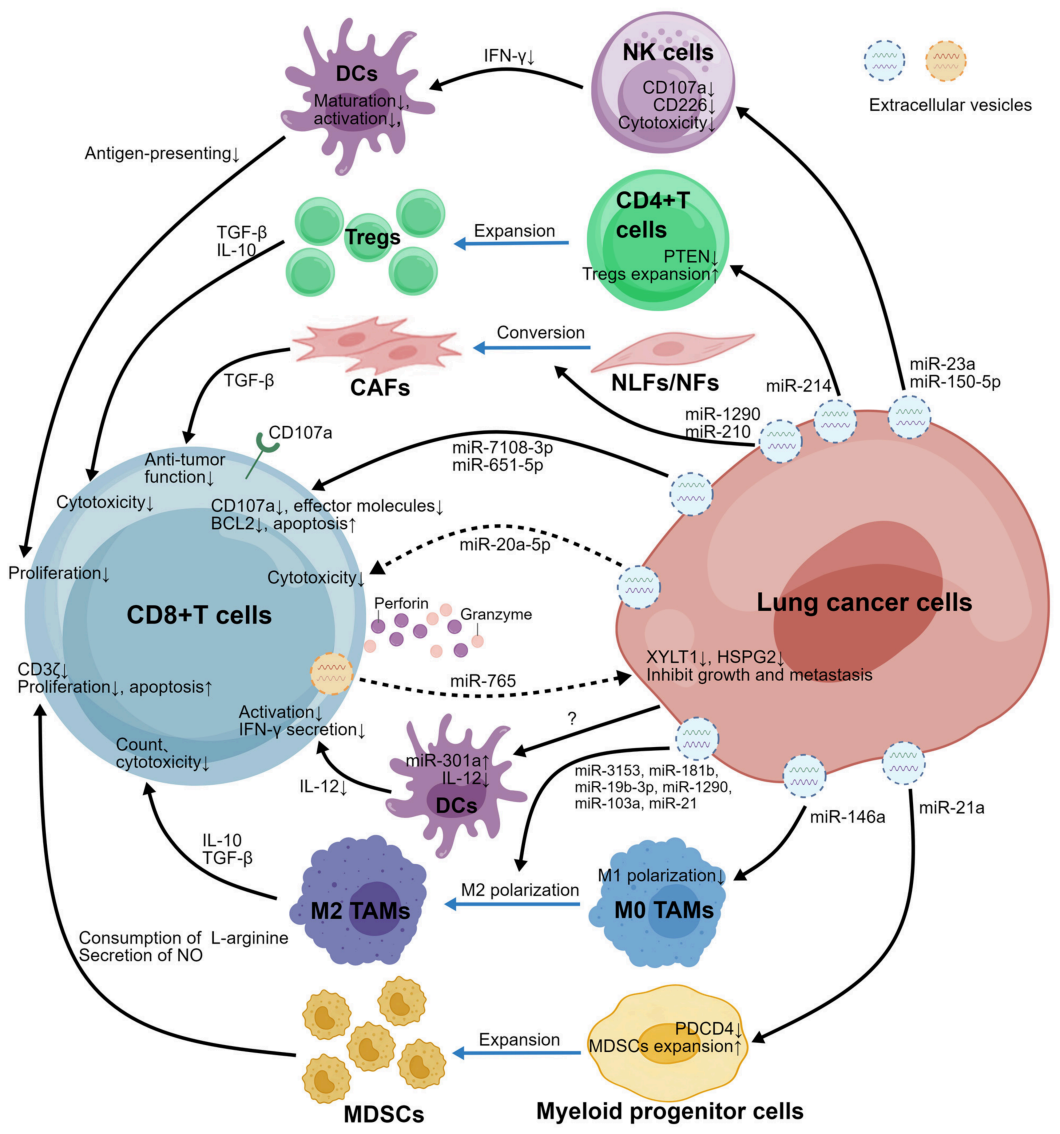
Schematic diagram of miRNA-mediated bidirectional communication between lung cancer cells and CD8^+^ T cells. ? indicates mechanisms or steps that have not yet been elucidated. Arrows indicate the direction of change: ↑ represents upregulation, increased secretion, or enhanced function; ↓ represents downregulation, decreased secretion, or impaired function. (Created with BioGDP.com [117]).

In summary, lung cancer cells can secrete EVs rich in specific miRNAs. These EVs not only act as “long-range weapons” to directly suppress the cytotoxic activity of CD8^+^ T cells, but also synergistically regulate cells in the TME—such as TAMs, Tregs, and MDSCs—thereby exerting a “relay-like” indirect suppression on CD8^+^ T cells through inhibitory factors secreted by these cells or highly expressed co-inhibitory ligands. Ultimately, this establishes an immunosuppressive barrier that accelerates malignant progression and immune evasion in lung cancer. At the same time, activated CD8^+^ T cells also possess the potential for reciprocal regulation and may transmit miR-765 via EVs to curb lung cancer progression. Consequently, an extremely complex and dynamic bidirectional communication network mediated by miRNAs has formed between lung cancer cells and CD8^+^ T cells (Figure 1).

### 5.4. Effects of Intracellular miRNA Dysregulation in Lung Cancer Cells on T Cell Function

#### 5.4.1. Effects of miRNA Dysregulation on the Remodeling of Intracellular Signaling and Soluble Factors

miRNAs can regulate intracellular signaling and paracrine networks in lung cancer cells, thereby influencing the antitumor function of CD8^+^ T cells. The miR-183/96/182 cluster can inhibit the progression and metastasis of NSCLC by promoting IL-2 secretion in NSCLC cells, which increases the enrichment of CD8^+^ T cells in the TME [118]. miR-760, on the other hand, can inhibit immune evasion in NSCLC by targeting and inhibiting indoleamine 2,3-dioxygenase 1 (*IDO1*) in NSCLC cells, thereby reducing CD8^+^ T cell apoptosis [119]. Furthermore, miR-505-3p enhances the cytotoxicity and cytokine secretion of co-cultured CD8^+^ T cells through direct cell–cell contact by specifically inhibiting neuroepithelial cell transforming gene 1 (*NET1*) in NSCLC cells [120]. However, the aforementioned miRNAs (miR-183/96/182 cluster, miR-760, and miR-505-3p) are downregulated in both NSCLC cells and tissues, suggesting that their expression deficiency is a key mechanism by which lung cancer achieves immune evasion. In summary, the dysregulation of the intrinsic post-transcriptional regulatory networks of lung cancer cells **is** critical to reshaping the TME and suppressing CD8^+^ T-cell activity. In this process, pathological downregulation or loss of key miRNAs in tumor cells can lead to paracrine dysregulation of related soluble cytokines or lift post-transcriptional repression of cytoplasmic metabolic enzymes and intracellular oncogenic signaling molecules, thereby weakening the immunological activity of CD8^+^ T cells.

#### 5.4.2. Remodeling of Co-Inhibitory Receptors and Antigen-Presenting Ligands on the Surface of Lung Cancer Cells

Lung cancer cells remodel cell surface ligands through aberrant expression of endogenous miRNAs, which is also a covert strategy they use to evade immune surveillance by CD8^+^ T cells. MHC-I presents specific antigenic peptides on the surface of tumor cells and is a key molecule mediating the recognition and elimination of tumor cells by CD8^+^ T cells [121]. Recent studies have shown that miR-149-5p, which is aberrantly upregulated in LUSC cells, can specifically inhibit NOD-like receptor family CARD domain-containing protein 5 (*NLRC5*), leading to a significant reduction in MHC-I molecules on the tumor cell surface [122]. This disruption of the antigen-presentation pathway helps tumor cells establish “immune camouflage” within the TME, enabling them to evade recognition and killing by CD8^+^ T cells.

Lung cancer cells also upregulate surface inhibitory ligands via miRNAs, thereby impairing the antitumor function of CD8^+^ T cells. CD155 on the surface of lung cancer cells can bind to the inhibitory receptor T cell immunoglobulin and ITIM domain (TIGIT) on the surface of CD8^+^ T cells, thereby inducing dysfunction of CD8^+^ T cells [121]. Research has demonstrated that in tumor tissues from LUAD patients, miR-326 expression is significantly negatively correlated with *CD155* expression, and miR-326 can negatively regulate *CD155* expression in LUAD cells [123]. Notably, miR-326 is markedly downregulated in NSCLC cells [124], suggesting that lung cancer cells derepress CD155 through the loss of this miRNA, thereby suppressing CD8^+^ T cell function via the CD155/TIGIT axis and driving immune evasion. CD276 is another co-inhibitory molecule highly expressed on the surface of tumor cells and is closely associated with poor patient prognosis. Although its receptor has not yet been identified, existing evidence supports that CD276 exerts a co-inhibitory role in the TME, suppressing the proliferation of CD8^+^ T cells and IFN-γ secretion [125]. Studies have shown that in clinical NSCLC tissue samples, miR-29a-3p expression is significantly negatively correlated with *CD276* expression, and it has been confirmed in human embryonic kidney cells (HEK293T cells) that miR-29a-3p can directly target and suppress *CD276* [126]. miR-29a-3p is expressed at low levels in NSCLC cells [127], suggesting that lung cancer cells relieve this post-transcriptional repression by downregulating miR-29a-3p, leading to aberrant overexpression of CD276 on the tumor cell surface and thereby suppressing CD8^+^ T cell-mediated antitumor immunity. Galectin-9, another key inhibitory ligand on the surface of tumor cells, binds to the TIM-3 receptor on the surface of CD8^+^ T cells, inhibits the secretion of effector cytokines, and promotes the exhaustion of CD8^+^ T cells [128]. Recent studies have confirmed that miR-491-5p specifically inhibits the expression of *LGALS9* (the gene encoding Galectin-9); however, gastric cancer cells can overcome this post-transcriptional repression by downregulating their own miR-491-5p, leading to the overexpression of Galectin-9 in tumor cells, which in turn suppresses CD8^+^ T cell function and promotes immune evasion [129]. miR-491-5p is typically lowly expressed in NSCLC cells [130], suggesting that lung cancer cells may lift the inhibition of Galectin-9 through the loss of expression of this miRNA, thereby allowing Galectin-9 to bind to TIM-3 on the surface of CD8^+^ T cells and suppress the antitumor function of CD8^+^ T cells. Unlike the accumulation of Galectin-9 caused by the loss of expression of the aforementioned tumor-suppressing miRNA, tumor cells can also induce the enrichment of Galectin-9 by highly expressing tumor-promoting miRNAs. Studies have shown that hepatocellular carcinoma cells can, through the upregulation of miR-93-5p, specifically inhibit inhibitor of growth family member 4 (*ING4*) and AE-binding protein 2 (*AEBP2*), thereby inducing the expression and secretion of Galectin-9, which leads to the inactivation of CD8^+^ T cells [131]. Given that miR-93-5p is also highly expressed in NSCLC cells [132], this suggests that lung cancer cells may induce the accumulation of Galectin-9 through the high expression of this miRNA, thereby suppressing the antitumor function of CD8^+^ T cells via the Galectin-9/TIM-3 axis.

In addition, tumor cells can also suppress the antitumor function of CD8^+^ T cells via the PD-L1/PD-1 axis [121], and specific miRNAs in the lung cancer microenvironment act as regulatory molecules that drive the aberrant accumulation of PD-L1 on the surface of tumor cells. For example, miR-20a can upregulate PD-L1 expression in NSCLC cells through two mechanisms: direct binding to *PD-L1* mRNA and targeted inhibition of *PTEN* [133]; miR-3127-5p promotes the phosphorylation of STAT3 by inhibiting autophagy in NSCLC cells, thereby also driving the accumulation of PD-L1 on the cell surface [134]. Furthermore, miR-20a [133] and miR-3127-5p [135] are all highly expressed in NSCLC cells. This suggests that NSCLC cells induce aberrant accumulation of PD-L1 on the cell surface by upregulating miRNAs that positively regulate PD-L1, thereby facilitating the binding of PD-L1 to PD-1 on the surface of CD8^+^ T cells, which in turn suppresses the antitumor activity of CD8^+^ T cells and ultimately promotes immune evasion in lung cancer. In contrast to the accumulation of PD-L1 expression caused by the aforementioned oncogenic miRNAs, some endogenous tumor-suppressive miRNAs exert a negative regulatory effect on PD-L1 expression. Studies have shown that let-7b can enter *Kras*-mutation-driven lung cancer cells and exert its tumor-suppressive effect by downregulating PD-L1 expression [15]; the miR-200 family, miR-34a, and miR-138-5p can also specifically target and inhibit PD-L1 expression in lung cancer cells [136,137,138]. However, let-7b [15], the miR-200 family [136], miR-34a [137], and miR-138-5p [139] are typically lowly expressed in lung cancer cells. This suggests that lung cancer cells lift the post-transcriptional repression of surface PD-L1 by downregulating miRNAs that negatively regulate PD-L1, thereby establishing a defense mechanism to evade immune killing by CD8^+^ T cells.

#### 5.4.3. Multidimensional Networks of Non-Coding RNAs Regulate Immune Checkpoints Through a Multi-Tiered Cascade

The aberrant accumulation of PD-L1 on the surface of lung cancer cells is also tightly regulated by an upstream competitive ceRNA network. Research has found that the lncRNAs *XIST*, *B4GALT1-AS1*, and *circ_0001006*, which are highly expressed in lung cancer cells, act as “molecular sponges” to bind and inhibit miR-34a-5p, miR-144-3p, and miR-320a, respectively. This lifts the post-transcriptional repression of PD-L1 exerted by these three miRNAs, leading to upregulation of PD-L1 expression [140,141,142]; upon binding to PD-1 on the surface of CD8^+^ T cells, elevated PD-L1 inhibits the secretion of IFN-γ and IL-2 by CD8^+^ T cells, thereby promoting immune evasion in lung cancer [140]. In addition to the aforementioned ceRNA axis that directly targets PD-L1, *circRNA-002178* exhibits a more complex dual regulatory mechanism. In LUAD cells, highly expressed circRNA-002178 acts as a “molecular sponge” to bind miR-34a-5p, thereby lifting its inhibition of PD-L1; simultaneously, *circRNA-002178* can be packaged into sEVs secreted by lung cancer cells and delivered to CD8^+^ T cells, where it binds to and inhibits miR-28-5p, leading to the upregulation of PD-1 on the surface of CD8^+^ T cells [143], which contributes to lung cancer immune evasion. This mechanism suggests that the same circRNA can synergistically establish an immune evasion barrier at both the tumor cell and immune cell ends. Furthermore, *circ_001678* and the lncRNA *FGD5-AS1*, which are highly expressed in NSCLC cells, can bind miR-326 and miR-454-3p, respectively, via a sponge mechanism, thereby upregulating zinc finger E-box binding homeobox 1 (*ZEB1*), increasing PD-L1 expression, thereby inhibiting the antitumor activity of CD8^+^ T cells and driving NSCLC immune evasion [144,145]; the latter can also simultaneously upregulate vascular endothelial growth factor A (*VEGFA*), promoting tumor angiogenesis [145]. Unlike the aforementioned oncogenic ceRNA axis, CircDENND2D is expressed at low levels in NSCLC cells. Its high expression can bind miR-130b-3p via a sponge mechanism, upregulate STK11, and suppress PD-L1 expression, thereby lifting the immunosuppression of CD8^+^ T cells by the PD-L1/PD-1 axis and inhibiting NSCLC metastasis and immune evasion [146], suggesting that the loss of CircDENND2D expression is a key mechanism by which lung cancer achieves immune evasion. This evidence indicates that immune evasion in the lung cancer microenvironment is not driven by a single miRNA, but rather is systematically regulated by a multidimensional crosstalk network consisting of lncRNA/circRNA-miRNA-mRNA within tumor cells, which is amplified through cascading interactions; ultimately, this complex regulatory network suppresses CD8^+^ T cell function via the PD-1/PD-L1 axis, providing potential multi-level targets for immune checkpoint-targeted intervention strategies.

Metabolic remodeling of the TME and hypoxia-induced miRNA responses are important but extensive topics beyond the scope of this review. We refer interested readers to recent comprehensive reviews on immunometabolic reprogramming and hypoxia-regulated miRNAs in lung cancer [147,148,149].

**Figure 2:**
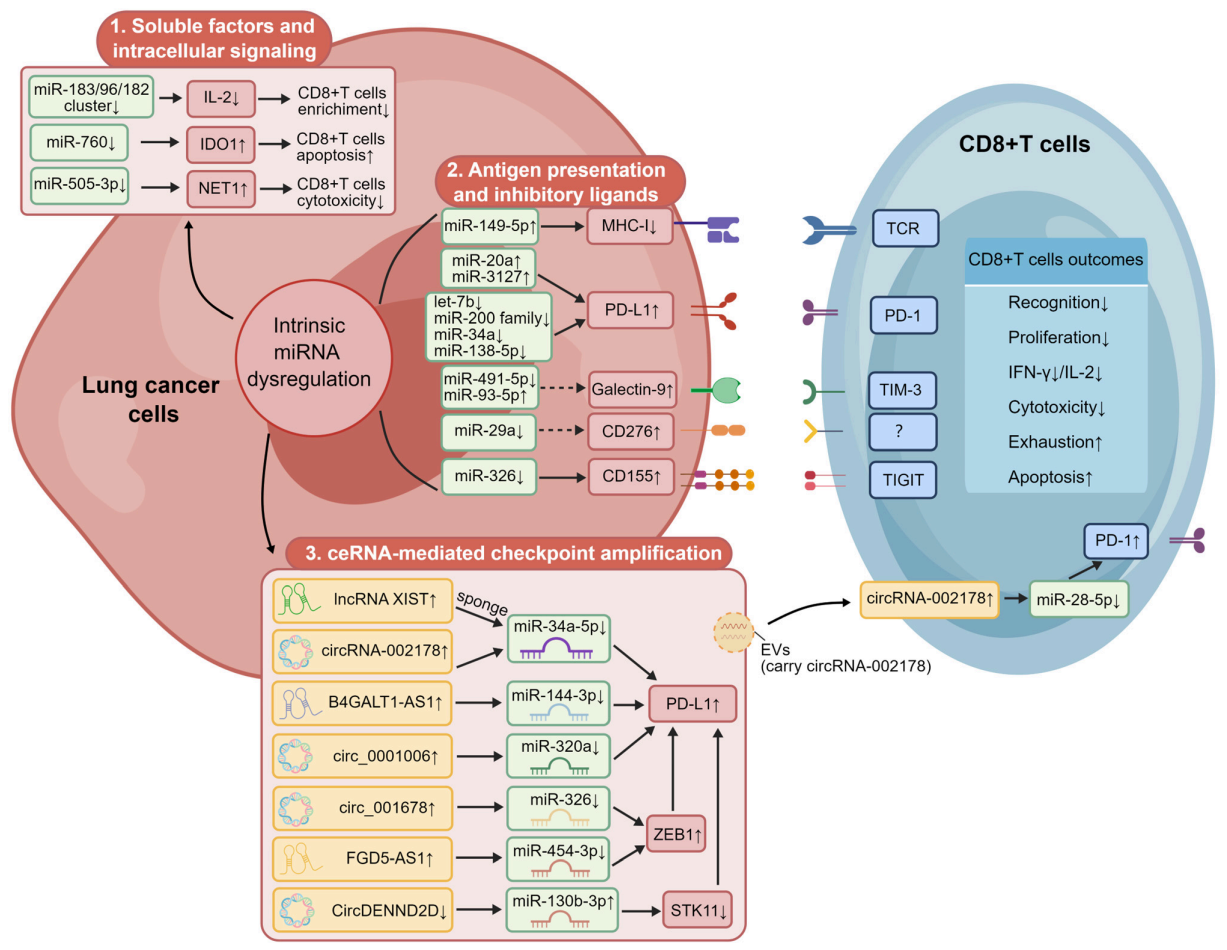
Schematic diagram of the molecular mechanisms by which endogenous miRNA dysregulation in lung cancer cells counteracts CD8^+^ T-cell killing. ? indicates an unidentified receptor. Arrows indicate the direction of change: ↑ represents upregulation, increased secretion, or enhanced function; ↓ represents downregulation, decreased secretion, or impaired function. (Created with BioGDP.com [117]).

In summary, lung cancer cells establish defense mechanisms against CD8^+^ T-cell killing at multiple levels through the coordinated action of several mechanisms—including aberrant expression of endogenous miRNAs, regulation of soluble factors, remodeling of intracellular pathways, upregulation of inhibitory ligands, and a multilevel ceRNA regulatory network composed of lncRNAs and circRNAs—thereby collectively attenuating the antitumor function of CD8^+^ T cells and facilitating immune evasion in lung cancer (Figure 2).

### 5.5. Effects of miRNA Expression Remodeling in the Lung Cancer TME on CD8^+^ T Cell Function

Based on the regulatory patterns of miRNAs on CD8^+^ T-cell cytotoxicity summarized in Table 1 above, and in combination with the expression characteristics of corresponding miRNAs in lung cancer tissues, these can be classified into two molecular patterns closely related to immune evasion, which correspond to the two core strategies employed by lung cancer to counteract CD8^+^ T-cell immune surveillance.

First, miRNAs that suppress CD8^+^ T-cell cytotoxicity—whose aberrantly high expression in lung cancer tissues or EVs constitutes a strategy by which tumors actively inhibit CD8^+^ T-cell cytotoxicity. For example, miR-23a, miR-301a, and miR-629-5p, as listed in Table 1, all function by blocking the secretion of effector molecules such as perforin, granzyme B, or IFN-γ through different targets. Studies have confirmed that this group of miRNAs, which suppress the cytotoxic activity of CD8^+^ T cells, is consistently and significantly overexpressed in lung cancer tissues or in EVs secreted by lung cancer cells [43,150,151]. Based on this, it is inferred that lung cancer cells suppress the cytotoxic activity of CD8^+^ T cells precisely by actively upregulating these immunosuppressive miRNAs, either through active secretion via EVs or via horizontal induction within the TME.

Second, for those miRNAs that enhance CD8^+^ T-cell cytotoxicity or reverse exhaustion, their aberrantly low expression in the lung cancer microenvironment constitutes a “covert escape” strategy employed by tumors to exploit the absence of tumor-suppressing signals. For example, let-7b, as shown in Table 1, promotes the secretion of IFN-γ and granzyme B; meanwhile, molecules such as miR-379-5p, miR-149-3p, and miR-27b-3p can downregulate immune checkpoint molecules such as PD-1, TIM-3, TIGIT, or TOX, thereby playing a key immunoprotective role in maintaining cytotoxic function and reversing CD8^+^ T cell exhaustion. However, in lung cancer tissues, these immunoprotective miRNAs are generally downregulated [152,153,154,155]. This suggests that lung cancer tissues achieve immune evasion precisely by downregulating this group of immunoprotective miRNAs, causing infiltrating CD8^+^ T cells to undergo functional exhaustion due to the loss of endogenous protection and the absence of activation signals.

In summary, the lung cancer microenvironment reshapes the regulatory network against CD8^+^ T-cell immune surveillance through the intertwined dual mechanisms of “high expression of immunosuppressive miRNAs” and “low expression of immunoprotective miRNAs.”

## 6. Conclusions and Outlook

Lung cancer cells employ a “two-pronged” immune evasion strategy against CD8^+^ T cells: namely, by aberrantly expressing endogenous miRNAs (such as miR-20a, miR-149-5p, and miR-326) to reshape their surface ligands (such as upregulating PD-L1, CD155, CD276, etc., and downregulating MHC-I) to establish “immune camouflage”; and by actively secreting EVs rich in miRNAs such as miR-7108-3p, miR-651-5p, they directly inhibit the function of CD8^+^ T cells. Furthermore, lung cancer cells can secrete specific miRNAs to reprogram cells in the TME—such as TAMs, NK cells, and MDSCs—thereby exerting a “relay-like” indirect suppression on CD8^+^ T cells through inhibitory factors secreted by these cells or co-inhibitory ligands they express. Activated CD8^+^ T cells also exhibit a counter-attack mechanism, which may involve secreting EVs rich in miR-765 to form a counter-regulatory network that restrains lung cancer progression. Furthermore, the ceRNA network composed of lncRNAs and circRNAs can regulate miRNA activity at multiple levels, further amplifying the immune evasion effect. In the future, in-depth analysis of this multidimensional miRNA-based crosstalk communication mechanism—and using it as a theoretical foundation to address the challenge of targeted vesicle delivery in the microenvironment—is expected to provide a highly promising new cell-free intervention approach to overcome current bottlenecks in lung cancer immunotherapy.

## Data Availability

No new data were created or analyzed in this study. Data sharing is not applicable to this article.

## References

[1] Bray F., Laversanne M., Sung H., Ferlay J., Siegel R.L., Soerjomataram I., Jemal A. (2024). Global cancer statistics 2022: GLOBOCAN estimates of incidence and mortality worldwide for 36 cancers in 185 countries. CA Cancer J. Clin..

[2] Inamura K. (2017). Lung Cancer: Understanding Its Molecular Pathology and the 2015 WHO Classification. Front. Oncol..

[3] Reck M., Rodríguez-Abreu D., Robinson A.G., Hui R., Csőszi T., Fülöp A., Gottfried M., Peled N., Tafreshi A., Cuffe S. (2021). Five-Year Outcomes with Pembrolizumab Versus Chemotherapy for Metastatic Non-Small-Cell Lung Cancer with PD-L1 Tumor Proportion Score ≥ 50. J. Clin. Oncol..

[4] Binnewies M., Roberts E.W., Kersten K., Chan V., Fearon D.F., Merad M., Coussens L.M., Gabrilovich D.I., Ostrand-Rosenberg S., Hedrick C.C. (2018). Understanding the tumor immune microenvironment (TIME) for effective therapy. Nat. Med..

[5] Shrestha C., Brito S., Deshar B., Cho J., Lee G., Jung D., Park J., Min K.S., Chae Y.K., Kim J. (2026). MicroRNA-regulated signaling and tumor microenvironment modulation in lung cancer: Mechanistic insights and translational opportunities. Cancer Cell Int..

[6] Zhao Y.-L., Li Y.-J., Wu C.-X., Xie N., Li X.-Y., Cao D.-C., Guo Y., Liang Y., Li Y.-M., Xue J.-N. (2025). m6A-modified circZNF548 regulates exosomal miR-7108-3p to activate CD3^+^CD8^+^ T cells and suppress NSCLC growth by JMY. BMC Biol..

[7] Zhao C., Cheng L., Li A., Wang H., Li X., Xu J. (2025). EGFR-Mutant Lung Adenocarcinoma Cell-Derived Exosomal miR-651-5p Induces CD8^+^ T Cell Apoptosis via Downregulating BCL2 Expression. Biomedicines.

[8] Zhou W.-J., Zhang J., Xie F., Wu J.-N., Ye J.-F., Wang J., Wu K., Li M.-Q. (2021). CD45RO^−^CD8^+^ T cell-derived exosomes restrict estrogen-driven endometrial cancer development via the ERβ/miR-765/PLP2/Notch axis. Theranostics.

[9] Su D., Wu G., Xiong R., Sun X., Xu M., Mei Y., Wu X. (2021). Tumor Immune Microenvironment Characteristics and Their Prognostic Value in Non-Small-Cell Lung Cancer. Front. Oncol..

[10] Xu Q., Hua X., Li B., Jiang B., Jin J., Wu R., Gu Y., Xu H., Cheng Q., Zhu S. (2025). Intrinsic STING of CD8^+^ T cells regulates self-metabolic reprogramming and memory to exert anti-tumor effects. Cell Commun. Signal..

[11] Tsurui T., Hosonuma M., Sasaki A., Maruyama Y., Amari Y., Funayama E., Tajima K., Toyoda H., Isobe J., Yamazaki Y. (2025). Ki-67 expression in anti-programmed cell death protein-1 antibody-bound CD8^+^ T cells as a predictor of clinical benefit. Discov. Oncol..

[12] Philip M., Schietinger A. (2022). CD8^+^ T cell differentiation and dysfunction in cancer. Nat. Rev. Immunol..

[13] Chowdhury D., Lieberman J. (2008). Death by a thousand cuts: Granzyme pathways of programmed cell death. Annu. Rev. Immunol..

[14] Hoekstra M.E., Vijver S.V., Schumacher T.N. (2021). Modulation of the tumor micro-environment by CD8^+^ T cell-derived cytokines. Curr. Opin. Immunol..

[15] Zhang Q., Pan J., Xiong D., Wang Y., Miller M.S., Sei S., Shoemaker R.H., Izzotti A., You M. (2021). Pulmonary Aerosol Delivery of Let-7b microRNA Confers a Striking Inhibitory Effect on Lung Carcinogenesis through Targeting the Tumor Immune Microenvironment. Adv. Sci..

[16] Lin R., Chen L., Chen G., Hu C., Jiang S., Sevilla J., Wan Y., Sampson J.H., Zhu B., Li Q.-J. (2014). Targeting miR-23a in CD8^+^ cytotoxic T lymphocytes prevents tumor-dependent immunosuppression. J. Clin. Investig..

[17] Chang X., Zhao J., Zhou Y., Guo M., Yan Y., Wang Y., Zhao X., Yang J., Chen C., Tang L. (2024). MiR-7 deficiency promotes Th1 polarization of CD4^+^T cells and enhances the antitumor effect in adoptive cell therapy for lung cancer. Immunol. Res..

[18] Cheng S., Li F., Qin H., Ping Y., Zhao Q., Gao Q., Song M., Qu J., Shan J., Zhang K. (2022). Long Noncoding RNA lncNDEPD1 Regulates PD-1 Expression via miR-3619-5p in CD8^+^ T Cells. J. Immunol..

[19] Aqeilan R.I., Calin G.A., Croce C.M. (2010). miR-15a and miR-16-1 in cancer: Discovery, function and future perspectives. Cell Death Differ..

[20] Wheeler B.D., Gagnon J.D., Zhu W.S., Muñoz-Sandoval P., Wong S.K., Simeonov D.S., Li Z., DeBarge R., Spitzer M., Marson A. (2023). The lncRNA Malat1 inhibits miR-15/16 to enhance cytotoxic T cell activation and memory cell formation. eLife.

[21] Wells A.C., Hioki K.A., Angelou C.C., Lynch A.C., Liang X., Ryan D.J., Thesmar I., Zhanybekova S., Zuklys S., Ullom J. (2023). Let-7 enhances murine anti-tumor CD8 T cell responses by promoting memory and antagonizing terminal differentiation. Nat. Commun..

[22] Chen S.-W., Zhu S.-Q., Pei X., Qiu B.-Q., Xiong D., Long X., Lin K., Lu F., Xu J.-J., Wu Y.-B. (2021). Cancer cell-derived exosomal circUSP7 induces CD8^+^ T cell dysfunction and anti-PD1 resistance by regulating the miR-934/SHP2 axis in NSCLC. Mol. Cancer.

[23] Li W., Han G., Li F., Bu P., Hao Y., Huang L., Bai X. (2024). Cancer cell-derived exosomal miR-20a-5p inhibits CD8^+^ T-cell function and confers anti-programmed cell death 1 therapy resistance in triple-negative breast cancer. Cancer Sci..

[24] Li X., Zhong M., Wang J., Wang L., Lin Z., Cao Z., Huang Z., Zhang F., Li Y., Liu M. (2019). miR-301a promotes lung tumorigenesis by suppressing Runx3. Mol. Cancer.

[25] Zhong G., Cheng X., Long H., He L., Qi W., Xiang T., Zhao Z., Zhu B. (2013). Dynamically expressed microRNA-15b modulates the activities of CD8^+^ T lymphocytes in mice with Lewis lung carcinoma. J. Transl. Med..

[26] Wissink E.M., Smith N.L., Spektor R., Rudd B.D., Grimson A. (2015). MicroRNAs and Their Targets Are Differentially Regulated in Adult and Neonatal Mouse CD8^+^ T Cells. Genetics.

[27] Yee Mon K.J., Kim S., Dai Z., West J.D., Zhu H., Jain R., Grimson A., Rudd B.D., Singh A. (2024). Functionalized nanowires for miRNA-mediated therapeutic programming of naïve T cells. Nat. Nanotechnol..

[28] Amado T., Amorim A., Enguita F.J., Romero P.V., Inácio D., de Miranda M.P., Winter S.J., Simas J.P., Krueger A., Schmolka N. (2020). MicroRNA-181a regulates IFN-γ expression in effector CD8^+^ T cell differentiation. J. Mol. Med..

[29] Cheng C.-C., Lin H.-C., Chiang Y.-W., Chang J., Sie Z.-L., Yang B.-L., Lim K.-H., Peng C.-L., Ho A.-S., Chang Y.-F. (2021). Nicotine exhausts CD8^+^ T cells against tumor cells through increasing miR-629-5p to repress IL2RB-mediated granzyme B expression. Cancer Immunol. Immunother..

[30] Zhong W., Luo N., Chen Y., Li J., Yang Z., Dong R. (2025). Exosomal Pparα derived from cancer cells induces CD8^+^ T cell exhaustion in hepatocellular carcinoma through the miR-27b-3p /TOX axis. Chin. Med. J..

[31] Lin Y.-Z., Liu C.-H., Wu W.-R., Liao T.-Y., Lee C.-C., Li H.-W., Chung F.-C., Shen Y.-C., Zhuo G.-Y., Liu L.-C. (2025). Memory-promoting function of miR-379-5p attenuates CD8^+^ T cell exhaustion by targeting immune checkpoints. J. Immunother. Cancer.

[32] Zhang M., Gao D., Shi Y., Wang Y., Joshi R., Yu Q., Liu D., Alotaibi F., Zhang Y., Wang H. (2019). miR-149-3p reverses CD8^+^ T-cell exhaustion by reducing inhibitory receptors and promoting cytokine secretion in breast cancer cells. Open Biol..

[33] Gigante M., Pontrelli P., Herr W., Gigante M., D’aVenia M., Zaza G., Cavalcanti E., Accetturo M., Lucarelli G., Carrieri G. (2016). miR-29b and miR-198 overexpression in CD8^+^ T cells of renal cell carcinoma patients down-modulates JAK3 and MCL-1 leading to immune dysfunction. J. Transl. Med..

[34] Ma J., Zhou Y., Chen J., Guo S., Zhang W., Yi X., Du P., Wang Y., Chen J., Li S. (2025). Exosomes enriched with miR-31-3p from keratinocytes under oxidative stress promote vitiligo progression by destructing melanocytes and activating CD8^+^ T cells. Int. J. Biol. Macromol..

[35] Yu T., Zuo Q.-F., Gong L., Wang L.-N., Zou Q.-M., Xiao B. (2016). MicroRNA-491 regulates the proliferation and apoptosis of CD8^+^ T cells. Sci. Rep..

[36] Chen Z., Stelekati E., Kurachi M., Yu S., Cai Z., Manne S., Khan O., Yang X., Wherry E.J. (2017). miR-150 Regulates Memory CD8 T Cell Differentiation via c-Myb. Cell Rep..

[37] He W., Wang C., Mu R., Liang P., Huang Z., Zhang J., Dong L. (2017). MiR-21 is required for anti-tumor immune response in mice: An implication for its bi-directional roles. Oncogene.

[38] Yin L.-B., Song C.-B., Zheng J.-F., Fu Y.-J., Qian S., Jiang Y.-J., Xu J.-J., Ding H.-B., Shang H., Zhang Z.-N. (2018). Elevated Expression of miR-19b Enhances CD8^+^ T Cell Function by Targeting PTEN in HIV Infected Long Term Non-progressors with Sustained Viral Suppression. Front. Immunol..

[39] Xie C., Wang S., Zhang H., Zhu Y., Jiang P., Shi S., Si Y., Chen J. (2023). Lnc-AIFM2-1 promotes HBV immune escape by acting as a ceRNA for miR-330-3p to regulate CD244 expression. Front. Immunol..

[40] Gracias D.T., Stelekati E., Hope J.L., Boesteanu A.C., Doering T.A., Norton J., Mueller Y.M., Fraietta J.A., Wherry E.J., Turner M. (2013). The microRNA miR-155 controls CD8^+^ T cell responses by regulating interferon signaling. Nat. Immunol..

[41] Ji Y., Fioravanti J., Zhu W., Wang H., Wu T., Hu J., Lacey N.E., Gautam S., Le Gall J.B., Yang X. (2019). miR-155 harnesses Phf19 to potentiate cancer immunotherapy through epigenetic reprogramming of CD8^+^ T cell fate. Nat. Commun..

[42] Ma F., Xu S., Liu X., Zhang Q., Xu X., Liu M., Hua M., Li N., Yao H., Cao X. (2011). The microRNA miR-29 controls innate and adaptive immune responses to intracellular bacterial infection by targeting interferon-γ. Nat. Immunol..

[43] Chandran P.A., Keller A., Weinmann L., Seida A.A., Braun M., Andreev K., Fischer B., Horn E., Schwinn S., Junker M. (2014). The TGF-β-inducible miR-23a cluster attenuates IFN-γ levels and antigen-specific cytotoxicity in human CD8^+^ T cells. J. Leukoc. Biol..

[44] Trifari S., Pipkin M.E., Bandukwala H.S., Äijö T., Bassein J., Chen R., Martinez G.J., Rao A. (2013). MicroRNA-directed program of cytotoxic CD8^+^ T-cell differentiation. Proc. Natl. Acad. Sci. USA.

[45] Li G., Chen W., Jiang K., Huang J., Zhong J., Liu X., Wei T., Gong R., Li Z., Zhu J. (2024). Exosome-mediated Delivery of miR-519e-5p Promotes Malignant Tumor Phenotype and CD8^+^ T-Cell Exhaustion in Metastatic PTC. J. Clin. Endocrinol. Metab..

[46] Mo Q., Yuan Z., Ning L., Wang G., Luo Q., Diao B. (2020). miR-199a-5p targeted regulation of MAGT1 expression in the functional depletion of CD8^+^T cells in HBV infection. Magnes. Res..

[47] Xiao C., Srinivasan L., Calado D.P., Patterson H.C., Zhang B., Wang J., Henderson J.M., Kutok J.L., Rajewsky K. (2008). Lymphoproliferative disease and autoimmunity in mice with increased miR-17-92 expression in lymphocytes. Nat. Immunol..

[48] Kosaka A., Ohkuri T., Ikeura M., Kohanbash G., Okada H. (2015). Transgene-derived overexpression of miR-17-92 in CD8^+^ T-cells confers enhanced cytotoxic activity. Biochem. Biophys. Res. Commun..

[49] Wu T., Wieland A., Araki K., Davis C.W., Ye L., Hale J.S., Ahmed R. (2012). Temporal expression of microRNA cluster miR-17-92 regulates effector and memory CD8^+^ T-cell differentiation. Proc. Natl. Acad. Sci. USA.

[50] Mitchell M.I., Loudig O. (2026). Extracellular MicroRNAs: A Stable and Diverse Source of Transcriptional Control. Biomolecules.

[51] Mathieu M., Martin-Jaular L., Lavieu G., Théry C. (2019). Specificities of secretion and uptake of exosomes and other extracellular vesicles for cell-to-cell communication. Nat. Cell Biol..

[52] Xu L., Wang L., Yang R., Li T., Zhu X. (2023). Lung adenocarcinoma cell-derived exosomes promote M2 macrophage polarization through transmission of miR-3153 to activate the JNK signaling pathway. Hum. Mol. Genet..

[53] Zhang X., Li F., Tang Y., Ren Q., Xiao B., Wan Y., Jiang S. (2020). miR-21a in exosomes from Lewis lung carcinoma cells accelerates tumor growth through targeting PDCD4 to enhance expansion of myeloid-derived suppressor cells. Oncogene.

[54] Yin Y., Cai X., Chen X., Liang H., Zhang Y., Li J., Wang Z., Chen X., Zhang W., Yokoyama S. (2014). Tumor-secreted miR-214 induces regulatory T cells: A major link between immune evasion and tumor growth. Cell Res..

[55] Blanchard N., Lankar D., Faure F., Regnault A., Dumont C., Raposo G., Hivroz C. (2002). TCR activation of human T cells induces the production of exosomes bearing the TCR/CD3/zeta complex. J. Immunol..

[56] Takenaka T., Nakai S., Katayama M., Hirano M., Ueno N., Noguchi K., Takatani-Nakase T., Fujii I., Kobayashi S.S., Nakase I. (2019). Effects of gefitinib treatment on cellular uptake of extracellular vesicles in EGFR-mutant non-small cell lung cancer cells. Int. J. Pharm..

[57] Hodakoski C., Hopkins B.D., Zhang G., Su T., Cheng Z., Morris R., Rhee K.Y., Goncalves M.D., Cantley L.C. (2019). Rac-Mediated Macropinocytosis of Extracellular Protein Promotes Glucose Independence in Non-Small Cell Lung Cancer. Cancers.

[58] Jin H.Y., Oda H., Chen P., Yang C., Zhou X., Kang S.G., Valentine E., Kefauver J.M., Liao L., Zhang Y. (2017). Differential Sensitivity of Target Genes to Translational Repression by miR-17~92. PLoS Genet..

[59] Hsin J.-P., Lu Y., Loeb G.B., Leslie C.S., Rudensky A.Y. (2018). The effect of cellular context on miR-155-mediated gene regulation in four major immune cell types. Nat. Immunol..

[60] Li Q.-J., Chau J., Ebert P.J., Sylvester G., Min H., Liu G., Braich R., Manoharan M., Soutschek J., Skare P. (2007). miR-181a is an intrinsic modulator of T cell sensitivity and selection. Cell.

[61] Essig K., Hu D., Guimaraes J.C., Alterauge D., Edelmann S., Raj T., Kranich J., Behrens G., Heiseke A., Floess S. (2017). Roquin Suppresses the PI3K-mTOR Signaling Pathway to Inhibit T Helper Cell Differentiation and Conversion of Treg to Tfr Cells. Immunity.

[62] Zhang Z., Li W., Jiang D., Gu L., Li B., Sang C., Rao D., Tang Z., Liu C. (2022). Silencing of long non-coding RNA linc01106 suppresses non-small cell lung cancer proliferation, migration and invasion by regulating microRNA-765. All Life.

[63] Zhang L., Meng Y., An Y., Yang X., Wei F., Ren X. (2024). The antitumor effect of extracellular vesicles derived from cytokine-activated CD8^+^ T cells. J. Leukoc. Biol..

[64] Wang W.-J., Wang J., Ouyang C., Chen C., Xu X.-F., Ye X.-Q. (2021). Overview of serpin B9 and its roles in cancer (Review). Oncol. Rep..

[65] Zhou Q., Wei S., Wang H., Li Y., Fan S., Cao Y., Wang C. (2023). T cell-derived exosomes in tumor immune modulation and immunotherapy. Front. Immunol..

[66] Seo N., Shirakura Y., Tahara Y., Momose F., Harada N., Ikeda H., Akiyoshi K., Shiku H. (2018). Activated CD8^+^ T cell extracellular vesicles prevent tumour progression by targeting of lesional mesenchymal cells. Nat. Commun..

[67] de Visser K.E., Joyce J.A. (2023). The evolving tumor microenvironment: From cancer initiation to metastatic outgrowth. Cancer Cell.

[68] Yamada K., Togashi Y. (2026). T-Cell Immunity and Lung Cancer. Respirology.

[69] Saeed A.F. (2025). Tumor-Associated Macrophages: Polarization, Immunoregulation, and Immunotherapy. Cells.

[70] Chen K., Luo L., Li Y., Yang G. (2025). Reprogramming the immune microenvironment in lung cancer. Front. Immunol..

[71] Petty A.J., Li A., Wang X., Dai R., Heyman B., Hsu D., Huang X., Yang Y. (2019). Hedgehog signaling promotes tumor-associated macrophage polarization to suppress intratumoral CD8^+^ T cell recruitment. J. Clin. Investig..

[72] Liu C.-Y., Wang Y.-M., Wang C.-L., Feng P.-H., Ko H.-W., Liu Y.-H., Wu Y.-C., Chu Y., Chung F.-T., Kuo C.-H. (2010). Population alterations of L-arginase- and inducible nitric oxide synthase-expressed CD11b^+^/CD14^−^/CD15^+^/CD33^+^ myeloid-derived suppressor cells and CD8^+^ T lymphocytes in patients with advanced-stage non-small cell lung cancer. J. Cancer Res. Clin. Oncol..

[73] Qin D., Zhang Y., Shu P., Lei Y., Li X., Wang Y. (2024). Targeting tumor-infiltrating tregs for improved antitumor responses. Front. Immunol..

[74] Huang S.W., Lai Y.G., Liao H.T., Chang C.L., Ma R.Y., Chen Y.H., Liou Y.H., Wu Z.Q., Wu Y.C., Liu K.J. (2025). Syngeneic natural killer cell therapy activates dendritic and T cells in metastatic lungs and effectively treats low-burden metastases. eLife.

[75] Deng S., Li Y., Peng L., Li J., Zhang L. (2026). Cancer-associated fibroblasts in non-small cell lung cancer therapy: Challenges and opportunities. Biochim. Biophys. Acta Rev. Cancer.

[76] Yan Q., Li S., He L., Chen N. (2024). Prognostic implications of tumor-infiltrating lymphocytes in non-small cell lung cancer: A systematic review and meta-analysis. Front. Immunol..

[77] Lim R.J., Salehi-Rad R., Tran L.M., Oh M.S., Dumitras C., Crosson W.P., Li R., Patel T.S., Man S., Yean C.E. (2024). CXCL9/10-engineered dendritic cells promote T cell activation and enhance immune checkpoint blockade for lung cancer. Cell Rep. Med..

[78] Marcovecchio P.M., Thomas G., Salek-Ardakani S. (2021). CXCL9-expressing tumor-associated macrophages: New players in the fight against cancer. J. Immunother. Cancer.

[79] Woods A.N., Wilson A.L., Srivinisan N., Zeng J., Dutta A.B., Peske J.D., Tewalt E.F., Gregg R.K., Ferguson A.R., Engelhard V.H. (2017). Differential Expression of Homing Receptor Ligands on Tumor-Associated Vasculature that Control CD8 Effector T-cell Entry. Cancer Immunol. Res..

[80] Sun X., Icli B., Wara A.K., Belkin N., He S., Kobzik L., Hunninghake G.M., Vera M.P., Blackwell T.S., Baron R.M. (2012). MicroRNA-181b regulates NF-κB-mediated vascular inflammation. J. Clin. Investig..

[81] Ma J., Chen S., Liu Y., Han H., Gong M., Song Y. (2022). The role of exosomal miR-181b in the crosstalk between NSCLC cells and tumor-associated macrophages. Genes Genom..

[82] Hart M., Nickl L., Walch-Rueckheim B., Krammes L., Rheinheimer S., Diener C., Taenzer T., Kehl T., Sester M., Lenhof H.-P. (2020). Wrinkle in the plan: miR-34a-5p impacts chemokine signaling by modulating CXCL10/CXCL11/CXCR3-axis in CD4^+^, CD8^+^ T cells, and M1 macrophages. J. Immunother. Cancer.

[83] Yi C., Zhang H., Guo Q., Lu L., Gao C., Zhao Y., Su Y., Lu J. (2025). MiR-4664-3p as a potential diagnostic, prognostic, and immunotherapeutic biomarker in NSCLC: Modulation of tumor progression through CD8^+^ T cell regulation. Front. Oncol..

[84] Dustin M.L. (2014). The immunological synapse. Cancer Immunol. Res..

[85] Yi M., Xu L., Jiao Y., Luo S., Li A., Wu K. (2020). The role of cancer-derived microRNAs in cancer immune escape. J. Hematol. Oncol..

[86] Li C., Qin F., Hu F., Xu H., Sun G., Han G., Wang T., Guo M. (2018). Characterization and selective incorporation of small non-coding RNAs in non-small cell lung cancer extracellular vesicles. Cell Biosci..

[87] Choi J.Y., Seok H.J., Lee D.H., Lee E., Kim T.-J., Bae S., Shin I., Bae I.H. (2024). Tumor-derived miR-6794-5p enhances cancer growth by promoting M2 macrophage polarization. Cell Commun. Signal..

[88] Xu L., Li K., Li J., Xu F., Liang S., Kong Y., Chen B. (2025). The crosstalk between lung adenocarcinoma cells and M2 macrophages promotes cancer cell development via the SFRS1/miR-708-5p/PD-L1 axis. Life Sci..

[89] Liu J., Fan L., Yu H., Zhang J., He Y., Feng D., Wang F., Li X., Liu Q., Li Y. (2019). Endoplasmic Reticulum Stress Causes Liver Cancer Cells to Release Exosomal miR-23a-3p and Up-regulate Programmed Death Ligand 1 Expression in Macrophages. Hepatology.

[90] Feng Y., Jin C., Wang T., Chen Z., Ji D., Zhang Y., Zhang C., Zhang D., Peng W., Sun Y. (2024). The Uridylyl Transferase TUT7-Mediated Accumulation of Exosomal miR-1246 Reprograms TAMs to Support CRC Progression. Adv. Sci..

[91] Berchem G., Noman M.Z., Bosseler M., Paggetti J., Baconnais S., Le Cam E., Nanbakhsh A., Moussay E., Mami-Chouaib F., Janji B. (2016). Hypoxic tumor-derived microvesicles negatively regulate NK cell function by a mechanism involving TGF-β and miR23a transfer. Oncoimmunology.

[92] Liu Z., Zhao W., Yang R. (2022). MiR-1246 is responsible for lung cancer cells-derived exosomes-mediated promoting effects on lung cancer stemness via targeting TRIM17. Environ. Toxicol..

[93] Yan Z., Wen J.-X., Cao X.-S., Zhao W., Han Y.-L., Wen X.-H., Yan L., Zhang M., Wang Y.-F., Hai L. (2022). Tumor cell-derived exosomal microRNA-146a promotes non-small cell lung cancer cell invasion and proliferation by inhibiting M1 macrophage polarization. Ann. Transl. Med..

[94] Hsu Y.-L., Hung J.-Y., Chang W.-A., Jian S.-F., Lin Y.-S., Pan Y.-C., Wu C.-Y., Kuo P.-L. (2018). Hypoxic Lung-Cancer-Derived Extracellular Vesicle MicroRNA-103a Increases the Oncogenic Effects of Macrophages by Targeting PTEN. Mol. Ther..

[95] Chen J., Zhang K., Zhi Y., Wu Y., Chen B., Bai J., Wang X. (2021). Tumor-derived exosomal miR-19b-3p facilitates M2 macrophage polarization and exosomal LINC00273 secretion to promote lung adenocarcinoma metastasis via Hippo pathway. Clin. Transl. Med..

[96] Jin J., Yu G. (2022). Hypoxic lung cancer cell-derived exosomal miR-21 mediates macrophage M2 polarization and promotes cancer cell proliferation through targeting IRF1. World J. Surg. Oncol..

[97] Gu J., Yang S., Wang X., Wu Y., Wei J., Xu J. (2023). Hypoxic lung adenocarcinoma-derived exosomal miR-1290 induces M2 macrophage polarization by targeting SOCS3. Cancer Med..

[98] Wu L., Zuo N., Pan S., Wang Y., Wang Q., Ma J. (2022). The Exosomal miR-1246 of Laryngeal Squamous Cell Carcinoma Induces Polarization of M2 Type Macrophages and Promotes the Invasiveness of Laryngeal Squamous Cell Carcinoma. J. Oncol..

[99] Wang Y., Chen W., Yu B., Wang L., Tang W. (2026). Exosomal miR-93-5p modulates macrophage polarization to enhance prostate cancer progression. Arch. Biochem. Biophys..

[100] He L., Chen Q., Wu X. (2025). Tumour-derived exosomal miR-205 promotes ovarian cancer cell progression through M2 macrophage polarization via the PI3K/Akt/mTOR pathway. J. Ovarian Res..

[101] Li N., Dhilipkannah P., Holden V.K., Sachdeva A., Jiang F. (2026). Red blood cell-derived exosomal miR-93-5p promotes lung cancer progression through PTEN suppression. Adv. Sci..

[102] Rabinowits G., Gerçel-Taylor C., Day J.M., Taylor D.D., Kloecker G.H. (2009). Exosomal microRNA: A diagnostic marker for lung cancer. Clin. Lung Cancer.

[103] Jiang M., Zhang W., Zhang R., Liu P., Ye Y., Yu W., Guo X., Yu J. (2020). Cancer exosome-derived miR-9 and miR-181a promote the development of early-stage MDSCs via interfering with SOCS3 and PIAS3 respectively in breast cancer. Oncogene.

[104] Qiu W., Guo X., Li B., Wang J., Qi Y., Chen Z., Zhao R., Deng L., Qian M., Wang S. (2021). Exosomal miR-1246 from glioma patient body fluids drives the differentiation and activation of myeloid-derived suppressor cells. Mol. Ther..

[105] Huang H., Wang Y., Gan J., Bu H., Li C., Li Y., Yang Y., Liu D. (2024). FRK exerts oncogenic effects by targeting NQO2 via exosomal miR-9-3p to regulate mitochondrial function. FASEB J..

[106] Chang W.-A., Tsai M.-J., Hung J.-Y., Wu K.-L., Tsai Y.-M., Huang Y.-C., Chang C.-Y., Tsai P.-H., Hsu Y.-L. (2021). MiR-150-5p-containing extracellular vesicles are a new immunoregulator that favor the progression of lung cancer in hypoxic microenvironments by altering the phenotype of NK cells. Cancers.

[107] Nakano T., Chen I.-H., Wang C.-C., Chen P.-J., Tseng H.-P., Huang K.-T., Hu T.-H., Li L.-C., Goto S., Cheng Y.-F. (2019). Circulating exosomal miR-92b: Its role for cancer immunoediting and clinical value for prediction of posttransplant hepatocellular carcinoma recurrence. Am. J. Transplant..

[108] Li M., Shan W., Hua Y., Chao F., Cui Y., Lv L., Dou X., Bian X., Zou J., Li H. (2021). Exosomal miR-92b-3p promotes chemoresistance of small cell lung cancer through the PTEN/AKT pathway. Front. Cell Dev. Biol..

[109] Tang B., Wu W., Wei X., Li Y., Ren G., Fan W. (2014). Activation of glioma cells generates immune tolerant NKT cells. J. Biol. Chem..

[110] Liu F., Peng W., Chen J., Xu Z., Jiang R., Shao Q., Zhao N., Qian K. (2021). Exosomes Derived from Alveolar Epithelial Cells Promote Alveolar Macrophage Activation Mediated by miR-92a-3p in Sepsis-Induced Acute Lung Injury. Front. Cell. Infect. Microbiol..

[111] Pyfferoen L., Mestdagh P., Vergote K., De Cabooter N., Vandesompele J., Lambrecht B.N., Vermaelen K.Y. (2014). Lung tumours reprogram pulmonary dendritic cell immunogenicity at the microRNA level. Int. J. Cancer.

[112] Zhou Z., Qu C., Zhou P., Zhou Q., Li D., Wu X., Yang L. (2024). Extracellular vesicles activated cancer-associated fibroblasts promote lung cancer metastasis through mitophagy and mtDNA transfer. J. Exp. Clin. Cancer Res..

[113] Fan J., Xu G., Chang Z., Zhu L., Yao J. (2020). miR-210 transferred by lung cancer cell-derived exosomes may act as proangiogenic factor in cancer-associated fibroblasts by modulating JAK2/STAT3 pathway. Clin. Sci..

[114] Zhou X., Yan T., Huang C., Xu Z., Wang L., Jiang E., Wang H., Chen Y., Liu K., Shao Z. (2018). Melanoma cell-secreted exosomal miR-155-5p induce proangiogenic switch of cancer-associated fibroblasts via SOCS1/JAK2/STAT3 signaling pathway. J. Exp. Clin. Cancer Res..

[115] Kewitz-Hempel S., Windisch N., Hause G., Müller L., Sunderkötter C., Gerloff D. (2024). Extracellular vesicles derived from melanoma cells induce carcinoma-associated fibroblasts via miR-92b-3p mediated downregulation of PTEN. J. Extracell. Vesicles.

[116] Ma Y., Wang D., Luo S., He Z., Sun J. (2022). Exosome miR-155-5p derived from lung cancer Hcc827 promotes macrophage activation and lung cancer progression. J. Biomater. Tissue Eng..

[117] Jiang S., Li H., Zhang L., Mu W., Zhang Y., Chen T., Wu J., Tang H., Zheng S., Liu Y. (2025). Generic Diagramming Platform (GDP): A comprehensive database of high-quality biomedical graphics. Nucleic Acids Res..

[118] Kundu S.T., Rodriguez B.L., Gibson L.A., Warner A.N., Perez M.G., Bajaj R., Fradette J.J., Class C.A., Solis L.M., Alvarez F.R.R. (2022). The microRNA-183/96/182 cluster inhibits lung cancer progression and metastasis by inducing an interleukin-2-mediated antitumor CD8^+^ cytotoxic T-cell response. Genes Dev..

[119] Ge H., Wang L., Chen W., Wang L. (2022). Mechanism of miR-760 Reversing Lung Cancer Immune Escape by Downregulating IDO1 and Eliminating Regulatory T Cells Based on Mathematical Biology. Comput. Math. Methods Med..

[120] Zhu P., Liu Z., Huang H., Zhong C., Zhou Y. (2020). MiRNA505/NET1 Axis Acts as a CD8^+^ T-TIL Regulator in Non-Small Cell Lung Cancer. OncoTargets Ther..

[121] Zhang B., Liu J., Mo Y., Zhang K., Huang B., Shang D. (2024). CD8^+^ T cell exhaustion and its regulatory mechanisms in the tumor microenvironment: Key to the success of immunotherapy. Front. Immunol..

[122] Ning B., Chiu D.J., Pfefferkorn R.M., Cullinane E., Kefella Y., Kane E., Reyes-Ortiz V., Liu G., Zhang X., Liu H. (2026). Upregulation of an Epithelial miRNA Is Associated with Immune Evasion in Progressive Bronchial Premalignant Lesions. Cancer Immunol. Res..

[123] Nakanishi T., Yoneshima Y., Okamura K., Yanagihara T., Hashisako M., Iwasaki T., Haratake N., Mizusaki S., Ota K., Iwama E. (2023). MicroRNA-326 negatively regulates CD155 expression in lung adenocarcinoma. Cancer Sci..

[124] Wang R., Chen X., Xu T., Xia R., Han L., Chen W., De W., Shu Y. (2016). MiR-326 regulates cell proliferation and migration in lung cancer by targeting phox2a and is regulated by HOTAIR. Am. J. Cancer Res..

[125] Picarda E., Ohaegbulam K.C., Zang X. (2016). Molecular Pathways: Targeting B7-H3 (CD276) for Human Cancer Immunotherapy. Clin. Cancer Res..

[126] Mei J., Luo Z., Cai Y., Wan R., Qian Z., Chu J., Sun Y., Shi Y., Jiang Y., Zhang Y. (2025). Altered Atlas of Exercise-Responsive MicroRNAs Revealing miR-29a-3p Attacks Armored and Cold Tumors and Boosts Anti-B7-H3 Therapy. Research.

[127] Zhang K., Han X., Hu W., Su C., He B. (2022). miR-29a-3p inhibits the malignant characteristics of non-small cell lung cancer cells by reducing the activity of the Wnt/β-catenin signaling pathway. Oncol. Lett..

[128] Wolf Y., Anderson A.C., Kuchroo V.K. (2020). TIM3 comes of age as an inhibitory receptor. Nat. Rev. Immunol..

[129] Huang W., Zheng W., Zhu D., Sun Z., Lu W., Tao J., Liu C., Li L., Zhou Y., Fan H. (2026). The LGALS9/miR-491-5p Axis Regulates CD8^+^ T Cell Function and Inhibits the Progression of Gastric Cancer. Cancer Sci..

[130] Wu F., Ji A., Zhang Z., Li J., Li P. (2021). miR-491-5p inhibits the proliferation and migration of A549 cells by FOXP4. Exp. Ther. Med..

[131] Dong Z.-R., Cai J.-B., Shi G.-M., Yang Y.-F., Huang X.-Y., Zhang C., Dong R.-Z., Wei C.-Y., Li T., Ke A.-W. (2023). Oncogenic miR-93-5p/Gal-9 axis drives CD8 (+) T-cell inactivation and is a therapeutic target for hepatocellular carcinoma immunotherapy. Cancer Lett..

[132] Yang W., Bai J., Liu D., Wang S., Zhao N., Che R., Zhang H. (2018). MiR-93-5p up-regulation is involved in non-small cell lung cancer cells proliferation and migration and poor prognosis. Gene.

[133] Gong J., Shen Y., Jiang F., Wang Y., Chu L., Sun J., Shen P., Chen M. (2022). MicroRNA-20a promotes non-small cell lung cancer proliferation by upregulating PD-L1 by targeting PTEN. Oncol. Lett..

[134] Tang D., Zhao D., Wu Y., Yao R., Zhou L., Lu L., Gao W., Sun Y. (2018). The miR-3127-5p/p-STAT3 axis up-regulates PD-L1 inducing chemoresistance in non-small-cell lung cancer. J. Cell. Mol. Med..

[135] Liu Y.-G., Li J., Nie F., Jin G.-W. (2022). LINC00961 functions as an anti-oncogene in non-small cell lung carcinoma by regulation of miR-3127. Am. J. Transl. Res..

[136] Chen L., Gibbons D.L., Goswami S., Cortez M.A., Ahn Y.-H., Byers L.A., Zhang X., Yi X., Dwyer D., Lin W. (2014). Metastasis is regulated via microRNA-200/ZEB1 axis control of tumour cell PD-L1 expression and intratumoral immunosuppression. Nat. Commun..

[137] Cortez M.A., Ivan C., Valdecanas D., Wang X., Peltier H.J., Ye Y., Araujo L., Carbone D.P., Shilo K., Giri D.K. (2016). PDL1 Regulation by p53 via miR-34. J. Natl. Cancer Inst..

[138] Zhang Q., Pan J., Xiong D., Zheng J., McPherson K.N., Lee S., Huang M., Xu Y., Chen S.-H., Wang Y. (2023). Aerosolized miR-138-5p and miR-200c targets PD-L1 for lung cancer prevention. Front. Immunol..

[139] Xing S., Xu Q., Fan X., Wu S., Tian F. (2019). Downregulation of miR-138-5p promotes non-small cell lung cancer progression by regulating CDK8. Mol. Med. Rep..

[140] Li J., Che L., Xu C., Lu D., Xu Y., Liu M., Chai W. (2022). XIST/miR-34a-5p/PDL1 axis regulated the development of lung cancer cells and the immune function of CD8^+^ T cells. J. Recept. Signal Transduct. Res..

[141] Huang L., Wu J., Sun Y., Yang Y., Liu Z. (2025). LncRNA B4GALT 1-AS1/miR-144-3p axis suppresses cytotoxicity of CD8^+^ T cells in lung adenocarcinoma by modulating PD-L1 expression. Discov. Oncol..

[142] Geng Z., Zhang G. (2025). circ_0001006 Promotes Immune Escape in Non-small Cell Lung Cancer by Regulating the miR-320a/PD-L1 Axis. Iran. J. Immunol..

[143] Wang J., Zhao X., Wang Y., Ren F., Sun D., Yan Y., Kong X., Bu J., Liu M., Xu S. (2020). circRNA-002178 act as a ceRNA to promote PDL1/PD1 expression in lung adenocarcinoma. Cell Death Dis..

[144] Tian Q., Wu T., Zhang X., Xu K., Yin X., Wang X., Shi S., Wang P., Gao L., Xu S. (2022). Immunomodulatory functions of the circ_001678/miRNA-326/ZEB1 axis in non-small cell lung cancer via the regulation of PD-1/PD-L1 pathway. Hum. Mol. Genet..

[145] Xia Y., Wang W.-C., Shen W.-H., Xu K., Hu Y.-Y., Han G.-H., Liu Y.-B. (2021). Thalidomide suppresses angiogenesis and immune evasion via lncRNA FGD5-AS1/miR-454-3p/ZEB1 axis-mediated VEGFA expression and PD-1/PD-L1 checkpoint in NSCLC. Chem. Biol. Interact..

[146] Chen Y., Chen X., Li Z., Zhu Y., Liu F., Cai J. (2023). CircDENND2D Inhibits PD-L1-Mediated Non-Small Cell Lung Cancer Metastasis and Immune Escape by Regulating miR-130b-3p/STK11 Axis. Biochem. Genet..

[147] Eivazzadeh Y., Orooji N., Eskandari T., Tarahomi M., Razavi F.T., Haghmorad D. (2026). Immunometabolism in lung cancer—The link between metabolism and immune response. iScience.

[148] Thangavelu L., Imran M., Alsharari S.H., Abdulaziz A.M., Alawlaqi A.M., Kamal M., Rekha M.M., Kaur M., Soothwal P., Arora I. (2024). Exploring hypoxia-induced ncRNAs as biomarkers and therapeutic targets in lung cancer. Pathol. Res. Pract..

[149] Wang Z., Guo H., Song Y., Wang A., Yan Y., Ma L., Liu B. (2025). Lung cancer tumor immune microenvironment: Analyzing immune escape mechanisms and exploring emerging therapeutic targets. Front. Immunol..

[150] Shi Y.-K., Zang Q.-L., Li G.-X., Huang Y., Wang S.-Z. (2016). Increased expression of microRNA-301a in nonsmall-cell lung cancer and its clinical significance. J. Cancer Res. Ther..

[151] Zhu L., Chen Y., Liu J., Nie K., Xiao Y., Yu H. (2020). MicroRNA-629 promotes the tumorigenesis of non-small-cell lung cancer by targeting FOXO1 and activating PI3K/AKT pathway. Cancer Biomark..

[152] Jusufović E., Rijavec M., Keser D., Korošec P., Sodja E., Iljazović E., Radojević Z., Košnik M. (2012). let-7b and miR-126 are down-regulated in tumor tissue and correlate with microvessel density and survival outcomes in non–small–cell lung cancer. PLoS ONE.

[153] Jiang Y., Zhu P., Gao Y., Wang A. (2020). miR-379-5p inhibits cell proliferation and promotes cell apoptosis in non-small cell lung cancer by targeting β-arrestin-1. Mol. Med. Rep..

[154] Qin B., Tang D., Zhang M. (2024). miR-149-3p targeting TMPRSS4 regulates the sensitivity to cisplatin to inhibit the progression of lung cancer. Biomol. Biomed..

[155] Sun Y., Xu T., Cao Y.-W., Ding X.-Q. (2017). Antitumor effect of miR-27b-3p on lung cancer cells via targeting Fzd7. Eur. Rev. Med. Pharmacol. Sci..

